# ‘Finding a relationship’: Conversations between mental health and social care staff, and service users about service users’ romantic relationships

**DOI:** 10.1371/journal.pmen.0000184

**Published:** 2025-05-08

**Authors:** Angelica Emery-Rhowbotham, Helen Killaspy, Sharon Eager, Brynmor Lloyd-Evans

**Affiliations:** Division of Psychiatry, University College London, London, United Kingdom; PLOS: Public Library of Science, UNITED KINGDOM OF GREAT BRITAIN AND NORTHERN IRELAND

## Abstract

Most people seek to establish romantic or intimate relationships in life, including people with mental health problems. However, this has been a neglected topic in mental health practice and research. This study aimed to investigate views of mental health and social care staff about the appropriateness of helping service users with romantic relationships, barriers to doing this, and suggestions for useful ways to support this. An online survey comprising both closed, multiple response and free-text questions was circulated to mental health organisations across the U.K. via social media, professional networks and use of snowballing sampling. A total of 63 responses were received. Quantitative data were analysed using descriptive statistics, and are reported as frequencies and percentages. Qualitative data were interpreted using thematic analysis, using an inductive approach. Although most participants reported that ‘finding a relationship’ conversations were appropriate in their job role, many barriers to supporting service users were identified, including: a lack of training; concerns about professional boundaries; concerns about service user capacity and vulnerability; and concerns about being intrusive. Participant suggestions for future support included educating service users on safe dating behaviours, and practical interventions such as assisting service users to use dating sites and engage with social activities to develop social skills and meet others. Staff were willing to help service users seek an intimate relationship but may need specific training or guidance to facilitate this confidently and safely. This study elucidates the need for further research in this area, particularly in understanding service user perspectives, and in developing resources to support staff in this work.

## Author summary

This study analysed 63 online survey responses completed by mental health and social care staff to understand their views around offering service users support to find a romantic relationship. We investigated whether mental health and social care staff think it is appropriate to talk to service users about their wishes for romantic and sexual relationships, what barriers get in the way of offering this support, and their ideas about what types of support might be possible and useful. Findings showed that most participants thought having conversations about romantic relationships were appropriate in their jobs. However, participants also described barriers to offering this support, including not having training on the topic, breaking professional boundaries, concerns about service users’ vulnerability and capacity to engage in a relationship, and concerns about seeming intrusive. The suggestions participants made to offer this support included educating service users on safe dating behaviours, and practical interventions such as assisting service users to use dating sites and engage with social activities to develop social skills and meet others. This study highlights the need for further research in this area, particularly in understanding service user perspectives, and in developing resources to support staff in this work.

## Introduction

Intimate relationships are a “central aspect of being human” [1] and a “fundamental human right for all” [2]. They affect our environment, quality of life, and without them, basic psychological needs remain unfulfilled [3]. Intimate relationships, as discussed in this work, encompass those distinct from platonic friendships in involving physical intimacy, sexual activity, or romantic love [4]. They may include both monogamous and non-monogamous relationships.

Social relationships are associated with many facets of psychological health, including feelings of self worth and self-esteem and low levels of depression, anxiety, and substance use [5–7]. The quality and satisfaction of an intimate relationship may have a particularly important impact on wellbeing due to potentially heightened positive emotions and cognitions [8]. For those with a mental health condition, evidence shows various benefits of an intimate relationship, including: providing companionship [9]; helping individuals to stay calm and relaxed [10]; providing emotional support [11]; instilling confidence [12]; and allowing sexual and emotional expression [13].

Moreover, loneliness is a common problem reported amongst individuals with mental health problems, which may increase one’s risk of both physical and mental health problems, and predict poor recovery for those with an existing mental health problem [14]. For instance, up to 40% of individuals with depression report feeling lonely most of the time [15] and their odds of feeling lonely are ten times that of the general population [16]. Loneliness has been conceptualised as having three dimensions: intimate, relational, and collective [17]. While the latter two indicate a lack of wider social networks such as friends and communities, intimate loneliness indicates a lack of a close emotional attachment, and not being able to share intimacy with another [18]. While having an intimate partner or spouse can reduce one’s level of intimate loneliness [19–21], loneliness interventions rarely, if ever, address an individual’s need or desire for an intimate relationship [22]. Thus, investigation into whether and how mental healthcare services could support people to achieve desired intimate relationships, and combat intimate loneliness, is warranted.

McCann and colleagues conducted a systematic review in 2019 which reviewed papers on mental health service users’ views on needs for support regarding sexuality and intimacy [3]. They found that sexual intimacy is often a neglected topic both by mental health practitioners and service users. Most studies discussing intimacy and mental illness, represented in this review and in the wider literature, are focused on sexual intimacy in populations with severe mental illness, and consistently find that mental health staff typically do not discuss people’s needs and wishes for intimacy and romantic relationships [23,24]. Furthermore, this limited research primarily focuses on sexual health and diminishing the risk of sexually transmitted diseases, and neglects the positive rewards of intimate relationships such as sexual pleasure, connection and commitment [25–27].

The limited literature that has focused on the wider experience of intimate relationships suggests that mental health service users may benefit from support to achieve such a relationship. Besides struggling with maintaining relationships - for reasons including chronic low relationship satisfaction [28], and a hesitancy to trust and be intimate with another [29] - those with mental illness consistently report struggling to establish an intimate relationship in the first place. Relatively few people with serious mental illness (15% in one study [30]) have romantic relationships when compared to the general population [3,31], despite 71% spontaneously identifying intimate relationships as facilitating recovery [30]. The gap between wanting a relationship, but not attaining one, may be partly explained by stigma [30,32]. Societally, those with mental illness are often heavily stigmatised, for instance in being rated as below average on several factors related to mate selection, such as social status, sexual desirability and personality [33–35]. Furthermore, those with mental illness are often aware of these stigmatising attitudes, and can even develop self-stigmatising attitudes also [36]. For instance, 66–89% of psychiatric outpatients agreed that most people ‘do not have an interest in having a romantic/ sexual relationship with someone who has a mental illness’ [37]. This can lead to active avoidance of intimate relationships [32], contributing to the finding that, globally, 40% of those with depression have intentionally avoided initiating a close relationship [38].

In addition to stigma, reasons for not seeking an intimate relationship include the direct effects of symptoms such as poor self-esteem and low motivation; medication side effects such as extinguished libido [39]; and lack of opportunity to meet potential partners, particularly while being an inpatient [30,40]. It is also important to note the potential damaging effect of intimate relationships for those with mental illness. For instance, existing mental health problems can increase one’s vulnerability to intimate partner violence [41], and relationship loss can have “devastating” effects such as further social losses, increased loneliness, and a regression in one’s recovery [21,22]. Embarking on an intimate relationship should therefore be done under careful consideration by the service user, with the opportunity for discussion and support from a mental healthcare provider.

Despite these obstacles to developing positive romantic and intimate relationships, however, service users recognise both “being lonely” and a “lack of a significant relationship” as being direct barriers to their recovery [30]. And, when asked about this directly, many service users express a desire for help in attaining an intimate relationship [40], despite this being rarely offered [42].

In recent years, the adoption of recovery-oriented approaches in mental health care has increased focus on empowering people to achieve a meaningful, fulfilling life alongside their mental health problems, rather than symptom eradication being the main focus [43]. This includes helping people to build relationships, which is explicitly included in policy in many health care systems. In the U.K. for example, The Care Act 2014 specifies developing and maintaining personal relationships as an eligible need for support [44].

Despite this, relationships of an intimate nature are not routinely discussed in clinical mental health settings, and have not often been the focus of research [29,37]. Limited literature that investigates this omission suggests that mental healthcare staff are ambivalent as to whether they should support service users in the domain of intimate relationships. While staff note that having such a relationship can aid the progression of treatment [45], they also reference several barriers to discussing this subject with service users. These include: a lack of professional training or skills; uncertainty about the appropriateness of such conversations; personal discomfort around discussing sex and intimacy; and organisational factors such as a lack of time and resources [23,46].

Due to these issues, intimate relationships tend to have low priority within mental healthcare [47,48] despite service users’ willingness and the recognition of its relevance to therapeutic goals [3,49]. However, unless their clinician broaches the subject, service users tend to feel that conversations about intimacy are “out of bounds” [49], except when relationships are abusive or potentially contributing to their illness [42,50]. Equally, mental health practitioners often believe that service users should lead on raising the subject of intimacy [45,51]. For instance, more than half of clinical psychologists describe “never” or “rarely” discussing issues of sexual problems and sexual health with their clients [52]. As reported by service users, this can result in the belief that the experience of intimacy is unattainable for them [53].

Little is known about how mental health services could best support service users seeking an intimate relationship. ‘Dating skills’ groups have been trialled and found acceptable [54,55], yet so far these have been limited to male participants with psychosis. There are several additional practical suggestions in the literature, regarding how mental health staff might help service users who are seeking a relationship, including: accompanying service users to social events where they can gain social skills and meet people [32,54]; coaching service users on the use of dating websites and apps [32]; as well as providing education to service users about relationships, and opportunities for peer support [56]. However, none of these examples have been widely implemented or evaluated.

### Rationale, aim and objectives of this study

Given the importance of intimate relationships in people’s lives; the specific barriers for people with mental health problems in finding relationships; and the general lack of knowledge about how to support service users in this domain, this study aimed to investigate: the perspectives of U.K. mental health and social care staff on supporting service users to find an intimate relationship; barriers to doing so; and suggestions as to how to increase support to enable this.

### Research questions

Due to the limited existing literature on this topic, the current study took an exploratory approach to address three research questions:

1. What are staff perspectives around the appropriateness of supporting service users with achieving desired intimate relationships?
2. What helps and hinders staff to have ‘finding a relationship’ conversations?
3. What strategies can staff use to support people with finding a relationship?

## Methods

### Ethics Statement

This study was approved by the UCL Research Ethics Committee on the 19^th^ May 2023 (Project ID: 24833/001). Formal consent was obtained from participants, who were provided information about the study at the start of the online survey, and clicked to agree to take part and proceed to the survey questions. Please see S1 Text for the participant information.

### Design

This study employed a cross-sectional, mixed-methods design, to collect both quantitative and qualitative data through an online survey. Quantitative data were collected using closed, multiple choice survey questions, and qualitative data were collected using open, free text response questions. The survey was disseminated to mental health and social care staff within the U.K. using snowballing sampling.

### Setting

This study comprised an online survey constructed using Qualtrics software [version XM, 2023]. Respondents were directed to a link to the survey which took around 15 minutes to complete.

### Participants

Mental health staff from any professional group were eligible to take part. This included clinically trained staff such as psychiatrists, psychologist nurses, social workers and occupational therapists, as well as non-clinically qualified staff such as support workers, peer workers and social care staff. Staff working in health services, Local Authorities or voluntary sector organisations were all eligible to take part, as long as they provided care and support to people with mental health problems. Participants were excluded if they worked in a specialist relationship counselling, sex therapy or gender identity service as the study wished to focus on general mental health and social care services.

### Materials

The online survey questionnaire began by asking the participant to provide demographic information including their age, gender and ethnicity, as well as information about how long and in what capacity they had worked in mental health services. The survey questionnaire then asked whether the participant helped service users to find a relationship and if so, how; any barriers and facilitators they experienced in having ‘finding a relationship’ conversations with service users; any training and other support they had received in this area; as well as their opinions on particular methods of helping service users to find relationships. The matrices for rating particular barriers, and methods of helping service users, were created referencing staff responses in a recent focus group study [40]. That study identified barriers to relationship support across four domains: service user, staff, service and society related barriers. Within staff related barriers, themes included: concern about giving right information/advice to service user; protecting service user vulnerability; not considered part of staff role; boundaries between staff and service user, not wanting to be overly personal. Our survey also included free text space for staff to report any other perceived barriers to these conversations. The full survey questionnaire is provided in Supporting Information File S1 Text.

### Procedures

The survey was open for 11 weeks (19^th^ May to 28^th^ July 2023). All participants provided their informed consent online, before proceeding to the survey questions. For recruitment, invitation emails were sent to a list of 28 organisations or leads of professional networks, to cascade to their membership, compiled using the professional networks of each member of the research team. This included national networks for social workers, psychiatrists and nurses, and national voluntary sector organisations providing mental healthcare. Invitations were extended to both individuals and organisational contacts, and each contact was asked to further disseminate the survey in turn, in a snowballing approach. Social media outreach included posting on the organisational X (formerly Twitter) accounts of the research team, and sharing on mental health spaces on Facebook, LinkedIn and Reddit.

Once participants had clicked on the invitation link to the survey, they first read through all participant information and data protection information and, if satisfied, then clicked a check box to confirm their consent. They then proceeded to complete the questionnaire on Qualtrics [version XM, 2023]. Participants were not required to provide their name or contact details, but before finishing, participants were given the option to provide their email address if they wished to receive a final report of the study, or to agree to be contacted about participation in future, related studies.

While recruitment was ongoing, data was stored within the Qualtrics programme, and once data collection had ended, the survey was deleted from Qualtrics and all survey data were moved to UCL’s secure online folders.

### Analysis

#### Quantitative.

For the 12 closed, multiple choice questions, descriptive statistics were analysed using *Microsoft Excel.* Participant demographic and job-related characteristics, as well as other quantitative, opinion-based questions were reported using frequencies and percentages. Descriptive statistics were calculated using the number of participants who answered a particular question as opposed to total participants who accessed the survey, to account for varying engagement across questions.

#### Qualitative.

For the free text responses, Braun and Clarke’s [57] thematic analysis approach was utilised. An inductive approach to thematic analysis was used, meaning analysis of themes and sub-themes was data-driven and not informed by a theoretical framework [58]. Due to the relatively unexamined nature of the research question, qualitative responses were also quantified in order to gain some perspective on the most prevalent views. There were seven distinct free text response questions from the survey. One question asked about perceived appropriateness of ‘finding a relationship’ support, four asked about barriers to offering this support, and two asked about methods and suggestions to increase such support. Analysis was led by AER and undertaken in six phases. First, data familiarisation involved reading over all participant responses. Second, these responses were coded into ‘meaningful’ units of text. The third phase entailed organising these codes into themes, i.e., codes which could fall under the same category were grouped together and given a label. Phases four and five involved reviewing and agreeing the labelling of themes and sub-themes and refining labels by consensus where appropriate. Co-authors HK, SE and BLE reviewed a selection of coded transcripts at this point and reviewed initial coding and preliminary themes, which were refined through team discussion. It was at this point that the seven original questionnaire items were merged into three themes, (appropriateness, barriers, and methods/suggestions). The final phase involved producing an analytical report (for a full breakdown of the analysis process, see Supporting Information File S2 Text).

### Reflexive statement

A researcher’s personal characteristics and positioning inevitably shapes their understanding and analysis of material, particularly within thematic analysis due to the heightened subjectivity of researcher-led generation of themes [59]. Thus, acknowledging one’s positioning using reflexive practice is vital as, being privy to the assumptions underlying the analysis, both author and reader are able to challenge them, protecting the work from undue bias [60]. In the current study, all co-authors are white, three are female, and all are educated to at least post-graduate level. The lead author has little experience of working with adults in a mental healthcare role. One co-author is a senior clinical academic psychiatrist and thus has relevant clinical experience, limiting misinterpretation of findings. Other co-authors include an early career researcher and a senior mental health academic with a background in social work. The lead author acknowledges a personal belief in the importance of offering intimate relationship support to service users, and therefore recognises the potential for the current paper to be written with a positive bias and an unduly critical response to staff’s reservations and perceived barriers to talking to service users about romantic and intimate relationships. To reduce subjectivity in this and other areas therefore, a coding diary was kept, which was used to reflect on personal responses to the literature, as well as participant responses which elicited an emotional response, or were felt to be puzzling. These reflections were discussed with the research team at regular intervals and informed the interpretation of the data.

## Results

### Participant characteristics: quantitative results

A total of 63 mental health and social care staff participated in the survey, and 44 completed all questions. The majority of respondents were female (n = 54), most were white (n = 49), in the age range 26–35 (n = 24), and did not follow a particular religion (n = 44). The number of years participants had worked in mental health services was relatively evenly split, with a small majority having worked for 2–5 years (n = 19). Most reported their profession to be psychologists (n = 16), who were working in the NHS (n = 46) in a community based mental health team (n = 32). See Table 1 for a full breakdown of participant characteristics, and see Supporting Information File S3 Text for all quantitative results tables. The full dataset of participants’ responses is provided in Supporting Information File S1 Data (with potentially identifying demographic characteristics removed).

**Table 1:** Participant characteristics.

Variables (Total *N* responses)		*n* (%)
Gender (*N* = 63)		
	Female	54 (85.7)
	Male	9 (14.3)
Age (*N* = 63)		
	18-25	12 (19.1)
	26-35	24 (38.1)
	36-45	15 (23.8)
	46-55	8 (12.7)
	56-65	4 (6.4)
	65+	0 (0)
Ethnicity (*N* = 63)		
	White	49 (77.8)
	Asian or Asian British	6 (9.5)
	Mixed or multiple ethnic groups	5 (7.9)
	Black, black British, Caribbean or African	2 (3.2)
Religion (*N* = 63)		
	No religion	44 (69.8)
	Christianity	10 (15.9)
	Buddhism	3 (4.8)
	Islam	3 (4.8)
	Hinduism	1 (1.6)
	Other	1 (1.6)
	Judaism	0 (0)
	Sikhism	0 (0)
Years working in mental health services (*N* = 60)		
	Less than 2 years	11 (18.3)
	2-5 years	19 (31.7)
	6-10 years	14 (23.3)
	More than 10 years	16 (26.7)
Professional group (*N* = 60)		
	Psychologist	16 (26.7)
	Occupational therapist	9 (15.0)
	Psychiatrist	9 (15.0)
	Support worker	9 (15.0)
	Nurse	6 (10.0)
	Peer support worker	3 (5.0)
	Social worker	3 (5.0)
	Counsellor/ therapist	2 (3.3)
	Other	2 (3.3)
Sector (*N* = 60)		
	NHS	46 (76.7)
	Independent sector	7 (11.7)
	Voluntary sector	5 (8.3)
	Local authority	0 (0)
Service type (*N* = 60)		
	NHS community mental health team	33 (55.0)
	Supported accommodation	10 (16.7)
	Inpatient	9 (15)
	Other	5 (8.5)

### Appropriateness of finding a relationship conversations: Quantitative results

Participants rated how far they agreed that ‘finding a relationship’ conversations were appropriate in their work role; 70% reported that they either ‘strongly’ or ‘somewhat’ agreed, and 8% ‘strongly’ disagreed (see Table 2).

**Table 2:** Ratings of agreement as to the appropriateness of ‘finding a relationship’ conversations.

Agreement as to appropriateness of ‘finding a relationship’ conversations	*n* (%) (total N = 50)
Strongly agree	10 (20.0)
Somewhat agree	25 (50.0)
Somewhat disagree	11 (22.0)
Strongly disagree	4 (8.0)

### Appropriateness of finding a relationship conversations: Qualitative results

A total of 45 participants responded to the free-text question of why they felt providing ‘finding a relationship’ support was, or was not, appropriate in their job role (see Table 3 below).

**Table 3:** Appropriateness of ‘finding a relationship’ support: Themes, sub-themes, example quotes and number of contributing participants.

Theme Subtheme	Illustrative quote [participant ID]	Number of participants who contributed to theme (total *N* = 45)
**Reasons for having ‘finding a relationship’ conversations**		**35/45**
Encouraging recovery	“I feel that romantic relationships and intimacy are a human need and have a huge impact on mental health” [ppt. 36]	15
Relevance to service users	“I believe this is important. Because many service users can struggle to form relationships in general, and having some support regarding intimate relationship would be useful for these users” [ppt. 58].	11
Methods to support service user relationships	“We should support our service users to recognise healthy relationships, build boundaries and encourage positive social interactions [ppt. 9]”	6
Implementing safe boundaries and limits	“I think as long as you keep it within certain boundaries, why shouldn’t you support them? [ppt. 22]”	5
**Reasons to not have ‘finding a relationship’ conversations**		**13/45**
Moral and ethical issues	“I feel like it would be unethical to help them find a relationship” [ppt. 3]	8
Not feasible in my job role	“Actually practically finding somebody feels a little beyond our scope” [ppt. 35]	5

Overall, 35 respondents expressed opinions around the reasons for having ‘finding a relationship’ conversations and for the most part, these were felt to be helpful in encouraging recovery. For some, this was mentioned in relation to a holistic model of practice, noting that staff should be working with “all aspects of life” [ppt. 48]. Otherwise, participants made an explicit link between having an intimate relationship and being better able to manage one’s mental health and loneliness.

Secondly, participants discussed how ‘finding a relationship’ conversations were generally relevant to service users. This was expressed by some in terms of particular service users, as they may “struggle with intimacy” [ppt. 26], or with “form [ing] relationships in general” [ppt. 58], rendering relationship seeking a relevant therapeutic goal. Others reflected that relationship attainment is desired by service users, and thus it is appropriate to “help/support/advise in ways I can” [ppt. 24].

Participants also reflected on specific methods to support service user relationships, and how these are particularly appropriate to engage in. Discussed here was the importance of supporting service users to recognise what is safe and acceptable in a relationship, as well as self-esteem building, skills building, and increasing social opportunities. Finally, some participants expressed the need for safe boundaries and limits within ‘finding a relationship’ conversations. For instance, one participant expressed that this work is appropriate “as long as you keep it within certain boundaries” [ppt. 33], with another expressing: “I think it’s only our work role if it’s [mental health] related, not just because someone without [mental health] can’t find a relationship” [ppt. 41]. (Note: both of these topics were discussed more completely later in the survey – see the ‘barriers’ and ‘methods and suggestions for support’ sections below for further discussion).

Fewer participants (13/45) expressed reasons not to engage in ‘finding a relationship’ conversations. Of those who did, eight discussed moral and ethical issues, as one stated directly: “I feel like it would be unethical to help them find a relationship” [ppt. 3].Respondents expressed a number of reasons for this. Some worried that ‘finding a relationship’ conversations might seem intrusive or impertinent to service users. Some noted that some service users were vulnerable to exploitation by sexual or romantic partners, or in some cases, might pose risks to others because of previous history of violence towards others. Staff expressed concerns that any conversation about service users’ wishes for romantic relationships could be construed as their encouragement for a particular choice or course of action, leading to professional culpability if something went wrong.

Some participants felt that ‘finding a relationship’ conversations were not feasible in their job role. This was for varying reasons, such as having a highly specified job description, where new goals could not be easily added to their agenda. Others noted a “lack of funding and commissioning” which limits staff to only offering interventions with clear “mental-health related outcomes” [ppt. 51]. Others discussed relying on other professionals such as “support workers” [ppt. 38] or “social workers/ occupational therapists” [ppt. 43], roles in which they believed ‘finding a relationship’ conversations may be more appropriate.

### Barriers to helping service users find a relationship: Quantitative data

Overall, participants had mixed opinions about which of the specified barriers were the most important. Rated the most important (highest rating for ‘a great deal’ of importance) was lack of training, followed by worries about professional boundaries, service user vulnerability, and intrusiveness. Some barriers were rated consistently as ‘not at all important’ by the majority, such as lack of management support, lack of time, and lack of training.

Lack of training and worries about professional boundaries were selected by some participants as highly important barriers, but were rated as unimportant barriers by other participants (see Table 4 below for a full outline of responses).

**Table 4:** Participant ratings of the importance of potential barriers to helping service users find intimate relationships.

Barrier	Importance rating			
	Not at all *n* (%)	A little *n* (%)	A moderate amount *n* (%)	A great deal *n* (%)
Lack of time (*N* = 48)	22 (45.8)	14 (29.2)	9 (18.8)	3 (6.2)
Inappropriateness (*N* = 47)	7 (14.9)	23 (48.9)	8 (17.0)	9 (19.2)
Intrusiveness (*N* = 48)	7 (14.6)	18 (37.5)	14 (27.1)	10 (20.8)
Triggering to service users (*N* = 48)	11 (29.9)	12 (25.0)	19 (39.6)	6 (12.5)
Worries about professional boundaries (*N* = 47)	14 (29.8)	10 (21.3)	12 (25.5)	11 (23.4)
Not feeling equipped to help (*N* = 47)	9 (19.2)	8 (17.0)	22 (46.8)	8 (17.0)
Service user vulnerability (*N* = 47)	5 (10.6)	19 (40.4)	13 (27.7)	10 (21.3)
Lack of management support (*N* = 47)	22 (46.8)	8 (17.0)	8 (17.0)	9 (19.2)
Lack of training (*N* = 47)	14 (29.8)	7 (14.9)	11 (23.4)	15 (31.9)

### Barriers to helping service users find a relationship: Qualitative data

Across three free text response questions, participants were asked to elaborate on any barriers that they perceived as being discouraging of ‘finding a relationship’ conversations. These are summarised in Table 5 below.

**Table 5:** Barriers to finding a relationship support: Themes, sub-themes, examples and number of contributing participants.

Theme Subtheme	Illustrative quote [participant ID]	Number of participants who contributed to theme (total *N* = 39)
**Staff factors**		**26/39**
Inappropriate for staff	“Feeling it is inappropriate in my work role” [ppt. 2]	15
Staff not feeling equipped to help	“Not being able to do anything to help if they want a relationship” [ppt. 59]	13
Communication issues	“The person who we support being able to properly communicate their desire for a relationship even if it is something they might want” [ppt. 13]	2
**Organisational factors**		**23/39**
Lack of support	“Lack of management support” [ppt. 5]	10
Lack of training	“I have experienced no training or discussions around this therefore naturally you think it might be out of the scope of your professional boundaries…” [ppt. 25]	10
Lack of policy	“Relationship goals not being part of routine assessment” [ppt. 46]	3
Lack of time and resources	“Time and appropriate services” [ppt. 12]	6
**Service user factors**		**14/39**
Relationships are inappropriate for the service user	“the individual may not be socially capable of a relationship, e.g., if they display traits of aggression” [ppt. 5]	14
Service user must be stable	“Sometimes people, at least in more acute services, might benefit from more stability (e.g., of mood, of routine, being able to go out) if they are then to find a good relationship.” [ppt. 51]	2
**External factors**		**5/39**
Societal perception	“the general perception of the public about people with mental illness dating” [ppt. 33]	3
Families being overprotective	“If the family are heavily involved, they can often be overprotective and not want their child to be dating” [ppt. 5]	3

In total, 23 of 39 respondents discussed staff factors as being notable barriers to ‘finding a relationship’ conversations, of whom 15 expressed ideas surrounding the perceived inappropriateness of relationship seeking support, especially due to feelings of intrusiveness and breaking professional boundaries. In addition, 10 respondents discussed not feeling equipped to help. Often this was due to fears about making things worse, for example “stigmatis [ing] [service users’] single status” [ppt. 40], or “com [ing] across as condescending” [ppt. 13]. Some participants expressed “not feeling able to help” [ppt. 57] or else having “low confidence” [ppt. 24] in matters related to relationships.

Approximately half (23) of the respondents also discussed organisational factors as a notable barrier. Ten described a lack of support, as one participant shared: “everything you do has to be mostly approved or encouraged by them, so … without management support, it is not something that can be done” [ppt. 5]. Ten participants reflected on the need for more staff training, as having “experienced no training or discussions around this … naturally you think it might be out of the scope of your professional boundaries” [ppt. 31]. Otherwise, six participants reported not having enough time or resources to focus on relationship conversations. As one participant said: “there is barely enough time to do the core aspects of my job, so … there is very unlikely to be resource for this” [ppt. 43].

Service user factors were cited by 14 of 39 respondents who answered this item. Here, participants expressed ideas regarding the inappropriateness of a relationship for some service users. Some worried about service users, being “vulnerable to exploitation” [ppt. 62], perhaps due to a history of “domestic violence” [ppt. 7], or “sexual assault” [ppt. 41]. Other participants perceived having a relationship would be detrimental to the service user, as it may “present another stressor…” [ppt. 2]. Moreover, some participants mentioned that a service user should be relatively stable before beginning to seek a relationship, as they may be more successful that way.

Finally, five participants mentioned external barriers. This included the families of service users being “overprotective” [ppt. 13], and not wanting them to engage in a relationship. Participants also mentioned societal pressures and perceptions as being barriers - either that people with mental illness should not be dating, or that staff should not perpetuate the societal pressure that everyone need be in a relationship to be happy.

### Nature of ‘finding a relationship’ conversations with service users: Quantitative data

The online survey questions asked participants to report on various aspects of their current practice regarding ‘finding a relationship’ conversations, including their perception of service user interest in finding a relationship. Overall, 64% reported that the majority of their service users were single, and 60% reported that the majority of their service users would not want to find a relationship.

Regarding ‘finding a relationship’ conversations specifically, 64% of respondents reported that they had had conversations with ‘few’ of their service users about finding a relationship, while a small proportion (10%) reported never having done so. Very few respondents reported ‘usually’ broaching the conversation (4%) and none said they ‘always’ brought the subject up. It was reported by most as easier to have ‘finding a relationship’ conversations in one-to-one settings compared to group situations, and finally only 7% reported having received any training on the topic of offering relationship support to service users (see Table 6 for a full breakdown of these findings).

**Table 6:** Quantitative findings regarding the nature of ‘finding a relationship’ conversations.

Variables		*n* (%)
Proportion of single service users (Total *N* = 50)		
	Less than 25%	6 (12.0)
	25-50%	12 (24.0)
	50-75%	18 (36.0)
	More than 75%	14 (28.0)
Proportion of single service users wanting to find a relationship (Total *N =* 50)		
	Less than 25%	14 (28.0)
	25-50%	16 (32.0)
	50-75%	9 (18.0)
	More than 75%	11 (22.0)
Proportion of service users ‘finding a relationship’ conversations are had with (Total *N* = 50)		
	None	5 (10.0)
	Few	32 (64.0)
	Many	10 (20.0)
	All, or nearly all	3 (6.0)
Frequency that ‘finding a relationship’ conversations are had (Total *N* = 50)		
	Never	5 (10.0)
	Rarely	26 (52.0)
	Sometimes	15 (30.0)
	Frequently	4 (8.0)
Who initiates ‘finding a relationship’ conversations (Total *N =* 50)		
	Always the service user	16 (32.0)
	Usually the service user	11 (22.0)
	Sometimes provider, sometimes service user	16 (32.0)
	Usually provider	2 (4.0)
	Always provider	0 (0.0)
	Not applicable	5 (10.0)
Preferred setting for ‘finding a relationship’ conversations (Total *N* = 57)		
	One to one	37 (64.9)
	Group	14 (24.6)
	Unsure	6 (10.5)
Participants who had training in ‘finding a relationship’ support (Total N = 46)		
	Training	3 (6.5)
	No training	43 (93.5)

### Support and suggestions offered to help service users find a relationship: Qualitative data

Participants were asked to suggest any methods that they had used to help service users with relationship seeking, as well as suggestions for methods to use in future practice. This item was answered by 37 respondents, and responses are summarised in Table 7 below.

**Table 7:** ‘Methods of Support’ themes, sub-themes, example quotes and number of contributing participants.

Theme Subtheme	Illustrative quote [participant ID]	Number of participants responses coded by theme and sub-theme (total *N* = 37)
**Current practice**		**33/37**
Preparing the service user for the dating world	“We try and sensitively explain how consensual relationships work. Remind them how coercive behaviour is wrong and try and guide them on acceptable behaviour.” [ppt. 7]	15
Discussions with service user about relationships	“Just having a conversation about it and finding out their thoughts.” [ppt. 33]	14
Increasing access to partners	“Behavioural experiments using dating apps and broadening social opportunities.” [ppt. 25]	5
**Suggestions for future practice**		**25/37**
Education and skills work	“Perhaps psycho [education] on healthy relationships, [and] for some people like LD services maybe learning social skills to develop relationships” [ppt. 47].	8
Increasing access to partners	“Social opportunities in services” [ppt. 11]	7
Systemic change	“Improving policies and access to material to meet needs” [ppt. 48]	6
Open discussion about relationships in service	“Open conversation. Looking at roles and routines that might support opportunities. We can explore what the emotional, practical and esteem barriers are.” [ppt. 49]	5
Signposting	“Direct them to relevant services” [ppt. 8]	3

Regarding current practice, 15 of 37 respondents described preparing the service user for the dating world. This method included educating the service user about dating safely, for instance, one shared that they: “provide support and encouragement, help people to identify their needs and goals, [and] teach people about communication and relationship skills” [ppt. 39]. Some respondents also described helping service users to build skills relevant to dating, such as: “building interpersonal social skills and recognising how they might be vulnerable to exploitation” [ppt. 10], or “building up self-esteem” [ppt. 11].

A total of 14 respondents shared thoughts about their discussions with service users. Discussion types were varied, for instance, some participants described “just having a conversation … and finding out their thoughts” [ppt. 33], while others had more directed conversations, for instance “to ask whether having a relationship is one of their goals” [ppt. 40]. Other discussions involved taking the service user’s lead, and discussing social connections, barriers, and sexual needs.

Increasing access to partners was discussed by five of 37 respondents. This involved directly supporting service users to use dating sites and apps, as well as helping to identify appropriate social opportunities.

Finally, four participants stated they were either “not sure” [ppt. 32] of current practice in their service, or that there was no relationship support provided: “In 20 years I’ve not witnessed this” [ppt. 34].

Suggestions for future practice generally reflected participants’ current practice. For instance, the most cited suggestion was education and skills work, which is in line with preparing the service user for the dating world, discussed above. Here, participants suggested increasing access to group support as a method to discuss “online dating safety” [ppt. 43], or to work through “scenarios [to] explore what they would do in [certain] situation [s]” [ppt. 30]. Some participants also recommended increasing the social skills requisite for developing an intimate relationship.

Seven of the 37 suggested increasing access to potential partners. For most, this meant helping service users to engage with social activities outside the service, e.g., “group activities in the community” [ppt. 4]. Others suggested “social opportunities in services” [ppt. 11], as well as “find [ing] dating services” [ppt. 31] either in person or online.

Respondents also suggested increasing discussion of relationships in the service (5/37). It was highlighted that such discussions be “more open… and not stigmatising [ppt. 33]”, and in such a way as “to give permission to patients to state this as a goal” [ppt. 40].

Finally, six respondents suggested systemic change, noting that mental health organisations need to be “improving policies and access to material to meet needs” [ppt. 48]. Three reported that signposting service users to other services is most appropriate.

## Discussion

The present study made several unique contributions to the topic of relationship seeking support in mental healthcare. Firstly, despite having had ‘few’ and ‘rare’ conversations about romance and intimacy with service users, the current participants mostly reported that ‘finding a relationship’ conversations were appropriate in their job role. Quantitative analysis showed that barriers to ‘finding a relationship’ conversations rated the most important were: a lack of training; concerns about professional boundaries; concerns about service user capacity and vulnerability; and concerns about being intrusive. Respondents reported having engaged in relationship seeking support through discussions with service users, and by taking steps to prepare them for entering the dating world. Participant suggestions for future support included educating service users on safe dating behaviours, and practical interventions such as assisting service users to use dating sites, and engage with social activities to develop social skills and meet others. This is one of very few studies to investigate mental health staff perspectives around the practice of helping service users to seek a relationship. Forrester-Jones and colleagues for instance [61] recently completed a small focus group study involving six mental health professionals, and similarly found that staff focused on the vulnerability of service users, risk, and their unease of discussing topics of intimacy. Our study corroborates these findings with a larger and more diverse sample of mental health and social care staff.

Amongst staff respondents, 70% agreed that ‘finding a relationship’ conversations were appropriate in their job role. One recent investigation of mental health practitioner attitudes found that only around 30% of participants expressed that dating and romantic relationships would be legitimate therapeutic goals [23]. These discordant results may be explained by cultural differences between the study settings (U.K. and Israel) and signal the need for more investigation on this topic. Although conducted with a small sample, all service user participants in one recent focus group simultaneously expressed the importance of having an intimate relationship, while lamenting the lack of support offered within mental healthcare [61], while in another focus group, service users rated support to find a romantic relationship as an important social inclusion need [40].

The barriers rated the most important were a lack of training, worries about professional boundaries, and worries about being intrusive. These findings suggest that respondents felt uncomfortable discussing issues related to personal relationships with service users. The concept of ‘boundaries’ in mental healthcare is nuanced and ‘crossing boundaries’ can be any combination of beneficial, neutral or harmful to the service user [62]; however some argue that the inflexible maintenance of professional boundaries perpetuates the power imbalance between service user and practitioner, and can be dehumanising to the former [63]. A series of nine reflective questions have thus been proposed to aid mental health practitioners considering a boundary crossing [64]. One of these is to imagine the ‘best possible outcome’ of crossing the boundary, and the ‘worst possible outcome’ both of crossing, and not crossing the boundary [64]. If relationship seeking support was promoted in mental healthcare, therefore, practitioners may be encouraged to reflect on and re-examine their professional boundaries in relation to this.

Similarly, when service users were asked about barriers, they cited an ambivalence towards asking for support, due to their romantic life being private, as well as an uncertainty as to whether – due to financial cuts – there would be time and space for substantial support [61].

Organisational factors were often rated to be unimportant barriers by the staff respondents. These findings diverge from existing literature. For instance, it is reported that a broad lack of guidance leaves mental healthcare staff with a perceived lack of competency, in turn leading to confusion, frustration and even low professional self-esteem [23]. A ‘lack of training’, however, was rated as being ‘not at all’ important and ‘very much’ important by a similar number of participants in the current study. These diverging views may reflect the difference in attitudes within the current sample. For the 30% of participants who disagree that ‘finding a relationship’ conversations are appropriate, they are likely to disagree that a lack of support or training is the key barrier to this, and agree that issues of appropriateness or professional boundaries are the key barrier. Future investigation, therefore, may benefit from stratifying analysis by participants who believe relationship seeking support is appropriate, and those who do not, such that nuances in perceptions may be explored.

Finally, service user factors were commonly discussed, where perceptions centred around service users either being too vulnerable, too volatile, or being in a context which made finding a relationship redundant. Service users themselves have reflected on the anxiety of seeking a relationship while managing symptoms of their illness and of their medication, managing self and social stigma, and the difficulty of having to disclose a diagnosis to a new partner [61]. A recent systematic review of the views and experiences of sexuality among people with severe mental illness [65] did not focus explicitly on service users’ needs for support, but concluded that both health service and community-level intervention is needed to reduce sexual stigma of mental illness and support service users’ needs for romantic and intimate relationships.

In the wider literature, staff attitudes that romantic and sexual relationships are either wholly ‘irrelevant’ or ‘detrimental’ to service users are discussed as paternalistic and potentially harmful to service user recovery [23]. The literature also cites that healthcare practitioners may assume that service users are ‘asexual’ [24,42,66], and relegate issues of sexual intimacy and romance to a position of low priority for this reason.

### Methods of relationship support: Current practice and suggestions

Staff respondents discussed a range of methods of support, including preparing the service user by educating them on dating safety, building relevant social skills, and direct methods of assistance such as dating app coaching or assisted socialising. In other social domains such as finding employment and housing, direct methods seem to have more support in the literature, and working firstly on a service user’s ‘readiness’ to engage is found to be unhelpful [67–70]. Moreover, regarding dating sites, it has been reported that using such online means makes it easier for those with mental illness to screen out inappropriate partners and locate a smaller pool of potential partners from a safe distance [32,71]. Furthermore, one documented skills group had accompanied social outings written into its manual, where service users were able to learn and develop social skills in a real dating environment, and afterwards discuss their experiences with their group leader, which service users found both acceptable and effective [54]. This literature tentatively suggests that direct methods of support may be preferable. However, due to a lack of literature comparing the effectiveness and acceptability of indirect compared to direct methods of support, this conclusion cannot be made without additional research.

The staff respondents also mentioned ‘discussions’ factoring in their current practice, and as a suggestion to improve future practice. In the literature, there exists some guidance surrounding the communication of sexual issues, which could potentially be extended to relationship seeking conversations also. For instance, both the PLISSIT [72,73] and BETTER [74] models advocate addressing topics of sexual wellness for disabled and ill patients in medical environments. These models have in common the following structure: (1) raising the topic of sex (2) explaining that sex is part of the quality of life (3) telling the service user about resources available, and (4) conveying their capacity in addressing concerns and questions [75]. Such a guided approach may have potential to help reduce staff hesitation about broaching this conversation.

### Strengths and limitations

A strength of this study is that all responses were provided anonymously and remotely. This means there was minimal social desirability bias, and participants could feel able to present any and all personal opinions without fear of judgement. Using an online survey as opposed to interviews meant there was more scope to hear from a wider range of staff working in a variety of roles in different mental health and social care services.

However, some limitations must also be acknowledged. Firstly, the typed, free-text question structure elicited fairly brief responses from participants. It was not possible to probe for more information, as would be the case within a semi-structured interview. This resulted in some responses being difficult to interpret.

Secondly, a small number of responses were received, due in part to the limited time period dictated by the lead author’s UCL Master’s programme deadline. This precluded any correlation analyses and/or inferential statistics to investigate associations between clinicians’ opinions, and the job roles and type of service that they worked in. We were unable to elicit any clear differences in views between groups of staff from different professional groups, firstly due to having a small sample, and secondly as demographic data were separated from participant responses in order to protect participant identity. Larger studies could inform whether certain roles or services need specific training, or more training than other roles or settings. The small sample size also means that the current results must be interpreted with some caution; those who were more interested in the topic may have been more likely to respond. This is also a limitation of convenience sampling methods such as snowballing. In addition, only 15% of respondents were male. Although this reflects the national gender proportions of staff working in mental health [76], there was some suggestion from our results that male staff may have felt less comfortable in having ‘finding a relationship’ conversations in general, and especially with female service users. We also had relatively small numbers of respondents from minoritised ethnic groups or from religious faiths other than Christianity, and so the perspectives of different cultural groups may not have been fully reflected in our findings. Finally, the current study did not investigate service user views on this topic, and therefore the perceived barriers and preferred methods of support may be different for service users. Further qualitative and quantitative studies are indicated, engaging with a broader range of stakeholders including service commissioners, and most importantly with service users – where their perspectives can be voiced and prioritised.

### Implications for practice

The first implication of the current study is the recognition of a need for acknowledgement and explicit support from mental health services and policy makers as to the importance of this topic. Clear and explicit guidance to staff about whether offering relationship seeking support is endorsed in the service is desirable. Services could also helpfully provide guidelines within service policy, alerting staff members to both what is expected of them regarding ‘finding a relationship’ conversations, as well as standard rules of conduct [24].

In addition to supportive policy, guidance or training for staff on the topic of supporting service users in regard to romance and intimacy is desirable. Training could address multiple issues raised by the current participants, such as discomfort, perception of inappropriateness and feelings of being ill-equipped. As suggested by the current participants, such training could increase knowledge about relationship and intimacy needs in mental illness, enhance skills to talk to service users about these topics comfortably, and most importantly, perpetuate the attitude that this is an integral aspect of psychological practice [49]. They would also address complex areas such as service user capacity and consent. Such training programmes may be informed by existing campaigns in the field of learning disabilities, such as *Supported Loving*, which educates both professionals and stakeholders on the importance of healthy romantic and sexual relationships for service users. The widespread success of this campaign underscores the equivalent need for such resources in the field of mental health [77].

## Conclusion

In conclusion, there appears to be willingness amongst mental healthcare staff to increase the provision of relationship seeking support to service users. While there is much hesitation, some of this may stem from the unfamiliarity of this topic area in mental health and social care services. Therefore, it is recommended that staff guidance, training programmes and ways of working are developed and evaluated. These should be informed by future in-depth qualitative research with service users and staff, to educate and improve staff confidence and skills on appropriate methods to support service users who wish to seek an intimate relationship. Such resources may empower mental healthcare staff to have open, non-biased discussions with service users, and to implement direct methods of relationship seeking support, such as supporting with accessing dating sites, social opportunities, and dating skills groups. Overall, this study has identified a need for more research and work in this area to encourage staff to have conversations with service users about finding intimacy, in the interest of achieving important, but currently neglected goals for service user recovery and quality of life.

## Supporting information

S1 Text Online survey and participant information documents. (DOCX)

S2 Text Coding process from free-text responses. (DOCX)

S3 Text Collated quantitative results tables. (DOCX)

S1 Data Anonymised dataset. (XLSX)
