# Supplementary material for: ‘Finding a relationship’: Conversations between mental health and social care staff, and service users about service users’ romantic relationships
Source: PLOS Ment Health. 2025 May 8;2(5):e0000184. doi: 10.1371/journal.pmen.0000184 (PMC12798258; doi:10.1371/journal.pmen.0000184)
Supplement: S1 Text — (DOCX) [file pmen.0000184.s001.docx]

**Supporting Information**

**S1 Appendix**

**Online survey including participant information documents**

Researcher contact details:

MSc Researcher: Angelica Emery-Rhowbotham; angelica.emery-rhowbotham.22@ucl.ac.uk

Principal investigator: Prof. Brynmor Lloyd-Evans; b.lloyd-evans@ucl.ac.uk.

Thank you for helping with this project which is being conducted by an MSc student in the Division of Psychiatry at UCL. Previous research at UCL has found that mental health service users who do not have an intimate / romantic relationship would often like to find a relationship but that staff and service users perceive some challenges with knowing how and when to talk about this and what help could be offered. The aim of this study is to learn from mental health and social care staff about how and when they talk to service users about finding a relationship, what barriers arise in discussing this, and suggestions for ways to help.

**Who are we looking for?**

We are seeking mental health and/or social care staff (whether working for the NHS, Local Authorities or voluntary sector services) to complete a brief online survey to tell us your views and experiences on this topic.

If you work in a specialist sexual health, relationship counselling, psychosexual therapy or Gender Identity service, please do not complete this survey. We are keen to understand the average occurrence of “finding a relationship” conversations in mental health settings. As conversations about relationships are well represented in the above settings, this study wishes to focus on settings where such conversations are less common.

**What happens if you agree to take part?**

If you decide to complete the survey, you will be asked a number of questions on your experience and opinions about helping service users to find a relationship. We will also ask you for some information about you: your age, gender, ethnicity and job role. The questionnaire will then ask you about: how you currently engage in helping service users to find a relationship; barriers and facilitators you experience in having “finding a relationship” conversations; training and other support you have been given in this area, as well as your opinions on particular methods of helping service users to find relationships.

These data will be kept anonymous with no way of tracing them to you. You can complete the survey anonymously, without leaving your name. This means that we as a research team will not know who has completed the questionnaire and who has not. However, if you wish, you can leave us your email address so the research team can send you a report of the results from the completed study, and, if you wish, contact you about taking part in a more in-depth interview in the future. If you decide to provide your email address for follow up and future contact, this will be stored securely and separately to your answers so that your data will remain anonymous.

The survey will take up to about 15 minutes to complete. You can quit the survey and leave it unfinished at any point if you wish and your responses will be deleted. However, please note if you would like to revisit the survey again, you will have to restart the questionnaire from the beginning. Because the data are anonymised, it is not possible to withdraw your data once you have completed the survey.

We hope this work may result in increased understanding of ways to help service users to find a relationship. We hope it may also increase understanding about what support mental health and social care staff need in having “finding a relationship” conversations and addressing service users’ needs for relationships.

Findings from this survey will be written up by the lead researcher for her MSc dissertation in September 2023, and will then be submitted for publication in a scientific journal. A report summarising findings from the study will be sent to all survey respondents who choose to leave a contact email.

This research project has been reviewed and approved by UCL REC. Ethics ID number: 24833/001.

**Local Data Protection Privacy Notice**

The controller for this project will be University College London (UCL). The UCL Data Protection Officer provides oversight of UCL activities involving the processing of personal data, and can be contacted at data-protection@ucl.ac.uk

This ‘local’ privacy notice sets out the information that applies to this particular study. Further information on how UCL uses participant information can be found in our ‘general’ privacy notice: please click here.

The information that is required to be provided to participants under data protection legislation (GDPR and DPA 2018) is provided across both the ‘local’ and ‘general’ privacy notices.

The categories of personal data used will be as follows:

Age

Gender

Ethnicity

Religion

Job title

How long you have worked in mental health or social care

Professional group

Type of service you work in

Type of sector you work in

The lawful basis that would be used to process your personal data will be performance of a task in the public interest.

The lawful basis used to process special category personal data will be for scientific and historical research or statistical purposes.

Your personal data will be processed so long as it is required for the research project. We will only be able to link these personal data to you if you choose to leave us an email address, otherwise the survey is completely anonymous at source. We will anonymise all personal data you provide us with, and will endeavour to minimise the processing of personal data wherever possible. Your email address (if you have chosen to provide it) will be kept securely for 12 months after your participation, at which point it will be destroyed. Other personal data will be archived for 10 years for the potential use of other researchers, at which point that too will be destroyed.

If you are concerned about how your personal data is being processed, or if you would like to contact us about your rights, please contact UCL in the first instance at data-protection@ucl.ac.uk.

**Contact for further information**

If you have any questions or concerns, please contact Angelica Emery-Rhowbotham (MSc researcher) at angelica.emery-rhowbotham.22@ucl.ac.uk or Prof. Brynmor Lloyd-Evans (Principal Investigator) at b.lloyd-evans@ucl.ac.uk.

If you feel that your concerns have not been adequately addressed or resolved by the research team, please escalate your concerns to UCL REC at ethics@ucl.ac.uk.

Please tick here to confirm you have read this information and consent to take part in this survey on this basis: ­­__

**Online survey**

Thank you for taking part in this study. What follows are some questions on the topic of relationships, specifically staff perspectives on “finding a relationship” conversations between mental health and social care workers and service users.

In “finding a relationship” conversations, a service user and staff member discuss the prospect of the service user finding an intimate / romantic relationship. A relationship may include a singular partner, or other type of dynamic such as a non-monogamous or polyamorous relationship. We are interested to discover the context and content of such conversations, as well as any barriers perceived by staff in having these conversations. The following questions should take up to 15 minutes to complete.

Please take care not to reveal any identifying details or personal information about service users you may be thinking of. This is in respect of their privacy.

After you have answered each question, press the 'next' button at the bottom of the screen to move to the next page. Please note once you have progressed to the next page, you will not be able to revisit or edit your previous answers.

Thank you for taking part in this survey.

Click the arrow to start the survey now. ->

Part 1 – About you.

1. How old are you?

- 18 - 25
- 26 – 35
- 36 – 45
- 46 – 55
- 56 – 65
- Over 65
- Prefer not to say

1. Which of the following best describes your gender?

- Male
- Female
- Other (please specify)
- Prefer not to say

1. Which of the following best describes your ethnicity?

Asian, Asian British / Black, Black British, Caribbean or African / Mixed or multiple ethnic groups / White / Other (please specify) / Prefer not to say

1. Do you follow a specific religion?

Buddhism / Christianity / Hinduism / Judaism / Islam / Sikhism / Other (please specify) / No religion / Prefer not to say

1. How long have you worked in mental health services?

Less than 2 years / 2-5 years / 6-10 years / More than 10 years / Prefer not to say

1. What is your current job title? (If you would rather not say, please leave this blank).

[Free text response]

1. Which professional group, if any, do you belong to?

Social worker / Occupational therapist / Psychologist / Psychiatrist / Counsellor, therapist / Support worker / Peer support worker / Other (please specify) / Prefer not to say

1. Which of the following, if any, do you work for?

NHS / Local authority / Voluntary sector organisation / Independent sector organisation / Prefer not to say

1. What sort of service do you mainly work in?

NHS community based mental health team / Day service (e.g. day centre, drop-in service, recovery college) / Supported accommodation service (e.g. residential service, supported housing, floating outreach) / Inpatient service / Other (please specify) / Prefer not to say

1. Do you work in the U.K. or elsewhere?

- U.K. / Elsewhere (please specify)

Part 2. Talking to Service Users

1. Some service users express that they would like help from services in finding and developing intimate / romantic relationships. How far do you agree that this is an appropriate aspect of your work role?

Strongly agree / Somewhat agree / Somewhat disagree / Strongly disagree

1. Please explain briefly why you think this is or is not part of your work role.

[Free text response]

1. Of the service users you support, how many are single? (Your best estimate is fine.)

Less than 25% / 25-50% / 50-75% / More than 75%

1. How many of these single service users would like to find a relationship? (Your best estimate is fine.)

Less than 25% / 25-50% / 50-75% / More than 75%

1. Of the service users you support who are single, with how many do you have conversations about “finding a relationship”?

None / Few / Many / All or nearly all

1. How often do you have “finding a relationship” conversations with the service users you support?

Never / Rarely / Sometimes / Frequently

1. Who initiates these conversations?
   - Always the service user
   - Usually the service user
   - Sometimes me sometimes them
   - Usually me
   - Always me
   - Not applicable – I don’t have these conversations

Part 3. Exploring barriers

1. How much do the following factors form barriers to “finding a relationship” conversations?

|  | **Not at all** | **A little** | **A moderate amount** | **A great deal** |
| --- | --- | --- | --- | --- |
| Lack of time |  |  |  |  |
| Feeling that it’s not appropriate to my work role |  |  |  |  |
| Worries about it feeling intrusive to the service user |  |  |  |  |
| Worries about it eroding professional boundaries |  |  |  |  |
| Worries about it being triggering for service users who have experienced abuse or sexual violence |  |  |  |  |
| Not feeling able to help if the person does want to find a relationship |  |  |  |  |
| Worries that the service user is vulnerable to exploitation and supporting them to find a relationship might not be in their best interest |  |  |  |  |
| Lack of management support |  |  |  |  |
| Lack of training |  |  |  |  |
| Other barrier (if applicable, please specify) |  |  |  |  |
| Other barrier (if applicable, please specify) |  |  |  |  |
| Other barrier (if applicable, please specify) |  |  |  |  |

1. Which of these barriers do you think is the most significant?

[Free text response]

1. Is there anything else you'd like to say about these barriers?

[Free text response]

1. In which context, if any, is it easier to have "finding a relationship" conversations?

- One-to-one conversations
- Group settings
- Other (please specify)
- Unsure

1. Please tell us if any of the following service user characteristics affect how comfortable you feel having “finding a relationship” conversations with service users? (please tick)

| **Gender** |  |
| --- | --- |
| **Religion** |  |
| **Age** |  |
| **Ethnicity** |  |
| **Sexuality** |  |
| **Level of social skills** |  |
| **Other (please specify)** |  |
| **Unsure** |  |

1. If you selected any of the factors above, please briefly explain how these affect how comfortable you feel having “finding a relationship” conversations.

[Free text response]

1. Please tell us about any ways in which you, or others you work with, try to help those who express a desire for an intimate / romantic relationship.

[Free text response]

1. Are there any other ways you think staff in mental health and social care services could support people in finding a relationship (even if these are not current practice in your workplace)?

[Free text response]

1. Have you been given any guidance or training in your current work about having “finding a relationship” conversations with service users?

- Yes / No

26a *If yes* Please tell us briefly about what guidance or training you have had regarding "finding a relationship" conversations.

[Free text response]

1. Are you aware of any organisations which provide examples of innovative practice in supporting service users who wish to find a relationship? If yes, please briefly tell us about them.

[Free text response]

1. How appropriate do you think it would be to provide the following types of support in your service? [Tick box matrix]

|  | **Not at all appropriate** | **Not very appropriate** | **Somewhat appropriate** | **Very appropriate** |
| --- | --- | --- | --- | --- |
| Listening and encouragement |  |  |  |  |
| Supporting service users to build confidence |  |  |  |  |
| Helping service users to access groups or activities that increase their social network |  |  |  |  |
| Supporting service users with social skills training |  |  |  |  |
| Helping service users to find resources for dating or meeting a potential partner |  |  |  |  |
| Helping service users to make an online dating profile |  |  |  |  |

1. How feasible do you think it would be to provide the following types of support in your service at the moment?

|  | **Not at all feasible** | **Not very feasible** | **Somewhat feasible** | **Very feasible** |
| --- | --- | --- | --- | --- |
| Listening and encouragement |  |  |  |  |
| Supporting service users to build confidence |  |  |  |  |
| Helping service users to access groups or activities that increase their social network |  |  |  |  |
| Supporting service users with social skills training |  |  |  |  |
| Helping service users to find resources for dating or meeting a potential partner |  |  |  |  |
| Helping service users to make an online dating profile |  |  |  |  |

Thank you for taking part in this study.

1. Would you like a report of the findings from this survey?

- Yes / no

1. May we keep your contact details to ask you if you would be interested in participating in any future research by our research group on this topic (e.g. an in-depth interview)?

- Yes / no

1. Could we contact you to ask about sending us a copy of your organisation’s policy on talking to service users about finding a relationship?

- Yes / no

If you answered yes to any of the above questions, please provide us with your email [by clicking on this link.](https://forms.gle/hCJ6jpgaDUnYQrtr8)
