# Supplementary material for: ‘Finding a relationship’: Conversations between mental health and social care staff, and service users about service users’ romantic relationships
Source: PLOS Ment Health. 2025 May 8;2(5):e0000184. doi: 10.1371/journal.pmen.0000184 (PMC12798258; doi:10.1371/journal.pmen.0000184)
Supplement: S2 Text — (DOCX) [file pmen.0000184.s002.docx]

**S2 Appendix**

**Coding process for free-text responses**

**Step 1: Creating initial themes for each of seven qualitative questions.**

***Question 1, Appropriateness:* ‘*Please explain briefly why you think this is or is not part of your work role.’***

AGREE

Part of holistic care or recovery

- Part of holistic care planning [6]
- Part of recovery and happiness [10]
- My patients talk a lot as part of their recovery is ‚’finding love’ [23]
- It's a key part of people's lives and recoveries. But does require some delicacy to do well and some evidence on how to do it well would be good too. [26]
- Relationships are an important aspect of overall wellbeing, and health. Whilst we may not be experts at supporting people in forming intimate relationships, or indeed matchmaking, our role should include an interest in all aspects of a person's emotional health - and they should be made to feel comfortable discussing such matters. Just as their physical wellbeing, or wishes regarding employment/education etc are all potentially relevant to their mental state. [30]
- its important for the individual's wellbeing, QoL etc. It can however be difficult to support, and in some instances need to considering safeguarding risks. [27]
- OTs work holistically with all aspects of life [36]

Identity or role fulfilment

- It is a vital part of identity and role fulfilment [14]

Service user’s desire

- Having a meaningful relationship with someone is many peoples goal. it is very much my role to help/support/advise in ways I can [15]
- this is a pressing concern and need in the service users I support [24]

Good for mental health

- Romantic relationships are key to managing mental health and to manage lonliness [16]
- I feel that romantic relationships and intimacy are a human need and have a huge impact on mental health. [25]

Recognising healthy relationships and how to behave in them

- but we should support our service users to recognise healthy relationships, build boundaries and encourage positive social interactions [9]
- To support them to think about what a healthy relationship is and to think about the way in which they meet new people. [19]

Self improvement support (indirect support)

- I would support with self-improvement goals to help the chances of finding a relationship e.g. healthy living, physical fitness, employment, hobbies [13]

Skills building helps therapeutic alliance

- however, helping them to develop appropriate skills would help to build therapeutic relationship and may result in better mental health outcomes for them. [3]

Only if MH related

- If they are struggling to maintain or find a relationship based on MH difficulties then i understand that our role can help them with self-esteem or any social anxiety but i think its only our work role if its MH related, not just because someone without MH cant find a relationship. Not sure how we are suppose to help there [29]
- I would see supporting someone to develop their skills and knowledge around navigating romantic relationships as a part of my role if important to the person. Particularly if mental health difficulties or learning difficulties or neurodivergence was a barrier to this.. [34]

Relevant therapeutic goal

- Patients are concerned regarding body image and struggle with intimacy [17]
- I think it should be more because it is a strong motivator and some patients do not have experience of developing relationships of any kind [32]
- Depression can be linked with loneliness and a lack of connection with others. Helping someone to try dating again could be really beneficial to their mental health. [33]

Service users want support

- I think people want this but do not express it as they dont feel it is within our role [18]

Considering needs and roles within relationship

- As a psychologist, I believe we have some capacity to help people to consider their needs and roles in relationships. [21]

Needs boundaries

- I think as long as you keep it within certain boundaries, why shouldn’t you support them? [22]
- Whatever the occupational barriers might be, i would explore goals and ways of overcoming them. There would be limits however. [37]

Help to access potential partners

- We can help people attend groups etc or meet new people but we can‚Äôt really promote anything that isn‚Äôt regulated. Would be a challenge to help them find this relationship [38]

DISAGREE

Unethical

- I feel like it would be unethical to help them find a relationship, [3]

Work role is closely delineated

- My work role involves logging into an app at the start of the shift, and ticking off the tasks that are listed e.g. prepare food, so the work is mostly set out for us. We do help assist with emotional support and distress, but no tasks are really geared towards building relationships. [5]
- ). I also do outreach as part of my job with an autistic male who is more high functioning, and has said once that he seeks a relationship. My role when I started was to encourage him to try new activities in London and discuss any emotional difficulties if they arise (such as struggling with depression), and I think that is the role I feel responsible for as opposed to helping them find a partner. [5]

Not something clients desire

- Additionally, the clients have severe mental/physical disabilities and so it is either not something they would desire (e.g. not mentally capable of), or [5]

Service users not capable of social interaction

- they are too severe to interact with the public/other society members e.g. prone to anger and attacking (e.g. not socially capable of) [5]

Not something service users bring up

- I think that is also how they view me so he wouldn’t necessarily go to me for that (possibly because they don’t deem as appropriate) unless I approached the topic. [5]

Abusive clients

- Many of the clients have history of being perpetrators of DV. [7]
- There are also risks to be considered, for example of people ending up in abusive relationships/one with very unequal power dynamics. [26]

Lack of time and resources

- Even though this is important, it is difficult to address these issues in the time slot given to me to see the service user. I also do not have access to resources or skills to be able to help someone like that. [8]
- Also, there is barely enough time to do the core aspects of my job, so in my opinion there is very unlikely to be resource for this. [31]

Can’t be matchmakers

- We cannot act as matchmakers [9]

Inappropriate

- I'm not sure how appropriate helping someone form an intimate relationship is? [12]
- It feels slightly intrusive to become involved in someone's romantic life; a breach of a professional boundary. [31]

Professional responsibility

- I would support with self-improvement goals to help the chances of finding a relationship e.g. healthy living, physical fitness, employment, hobbies, but not with actually finding a relationship as there is a concern about professional responsibility if something goes wrong

Safeguarding risks

- It can however be difficult to support, and in some instances need to considering safeguarding risks. [27]

Relying on other professionals

- tend to rely on support workers to help them socialise but can suggest this as part of the care plan [28]
- However, perhaps social workers / OTs within my team could run groups on safety / appropriate when online dating etc. which would be in keeping with this aim. [31]

Relationships are relevant, but not actually finding a partner

- It’s relevant to mental health, and somewhat relevant in terms of values and behaviour, but actually practically finding somebody feels a little beyond our scope [35]

IT DEPENDS

Depends on their level of care: high support not priority

- It depends on the level of care they require, in the more independent housing I would say I would be happy to discuss this but in high support this is not our priority [4]

Undecided

- I'm undecided I think it could be good for residents to find meaningful relationships but I wonder if it crosses a boundary to support them into those relationships [20]

***Question 2, Barriers:* ‘*How much do the following factors form barriers to “finding a relationship” conversations? - Other barrier (if applicable, please specify) – Text’***

Relationship being detrimental to the service user

- it is probable that a relationship may present another stressor to the service user's life (moderate amount) [4]

Service user not capable of relationship

- A view that the individual may not be socially capable of a relationship e.g. if they display traits of aggression (mod; 7)

Service user may put their partner at risk

- The clients who have asked are male and have a history of perpetrators of domestic violence [9]
- Might be a DV perpetrator themselves so this comes with risks [31]

Service user not asking for support

- The person who we support being able to properly communicate their desire for a relationship even if it is something they might want [17]

Service user not ready for a relationship

- just had a baby, might not be ready yet. [36]

Liability

- Worry that if it goes wrong will the resident blame me? [37]

Getting it wrong

- If a person has a severe learning disability and has always been single, conversations about relationships (if handled incorrectly)may come across as condescending or as if you are making fun of them [17]

Being ill equipped

- Also feel inequipped like it would be opening a tin of worms [50]

Other people are better suited to this work

- I think I'm not necessarily the best person as a consultant psychiatrist carrying out relatively formal reviews - conversations better initiated by people like care coordinators or support workers in a relatively informal setting. [44]

Not a priority issue

- Other management priorities [50]
- Needing to prioritise more pressing current issues (e.g. risk, accommodation, getting out more in geberal). Sometimes other things need to be in place first. [51]

Related barriers

- Other support around the person such as family or services being opposed [52], a little
- service users' previous relationship problems [4]

Colleague hesitation

- Colleagues opinions of whether it's our role [55]

Service organised differently

- Relationship goals not being part of routine assessment [52]

Not appropriate context

- starting up relationship problems in a therapeutic session [4]

Families being overprotective

- If the family are active in the person we supports lives, they could be overprotective and not want their child to have a relationship e.g. they feel they are too vulnerable or would not want staff to be involved in that way [17]
- If the family are heavily involved, they can often be overprotective and not want their child to be dating [7]

Needing therapeutic alliance first

- People not wanting to discuss this until they have built a trusting relationship with the professional [52], mod

Stigmatising singlehood

- also concerns that the discussion (where initiated by clinician) stigmatise their single status) [46]

***Question 3, Barriers: ‘Which of these barriers do you think is the most significant?’***

Inappropriate in my work role

- feeling it is inappropriate in my work role [P2]
- Worries that is inappropriate and intrusive [P4]
- Concerns re: relevance/appropriateness - not personally, but at service level and service user level. [P51]

Breaking boundaries

- Worries about it breaking professional boundaries [P9]
- Ensuring the support remains boundaries and the service user takes the lead if this is what they wish to do [P10]
- professional boundaries [P17]
- Professional boundaries, [P19]
- The barrier around professional boundaries [P31]
- crossing professional lines [P35]
- Boundaries [P51]
- Boundaries, triggering and intrusiveness [P58]

Intrusive

- Worries that is inappropriate and intrusive [P4]
- worries it might seem too intrusive [P41]
- Being intrusive to the service user. Also, a make worker asking a female might be viewed dimly or inappropriate. [P49]
- Boundaries, triggering and intrusiveness [P58]

Gender issues between client and clinician

- Being intrusive to the service user. Also, a make worker asking a female might be viewed dimly or inappropriate. [P49]
- Nature of the service and being a male member of staff I would not feel comfortable discussing with a female patient and would also be concerned that this would make them uncomfortable. [P54]
- I think gender of staff member [P54]

Relationships not prioritised

- Prioritising other issues [P45]

Lack of training

- Lack of training [P32]
- Lack of management training [P13]
- I have experienced no training or discussions around this therefore naturally you think it might be out of the scope of your professional boundaries. Something about it does feel that way. [P25]
- Lack of training around conversations and how these can be appropriate [P36]
- Lack of training perhaps [P42]
- Lack of training [P47]

Hard to identify clients

- Hard to identify just one and varies from person to person [P38]

Lack of time and resources

- Time [P8]
- Time and appropriate services [P12]
- Time, and feeling unable to help. [P43]
- Time constraints are a factor [P27]

Low clinician confidence

- voulnerability. I'm and experienced nurse although low confidence with this [30]
- not knowing how to support someone to seek a relationship and worry about their vulnerability [P24]
- Time, and feeling unable to help. [P43]
- Not feeling able to help [P57]
- Not being able to do anything to help if they want a relationship [P59]

Client’s history

- Client’s history of domestic violence [P7]
- Sexual assault [P41]

Client context

- Either there pregnant or have a baby up to the age of 2. May need support for this before getting into a new relationship. [P30]

Vulnerable patients

- vulnerable patients [P19]
- voulnerability. I'm and experienced nurse although low confidence with this [P24]
- not knowing how to support someone to seek a relationship and worry about their vulnerability [P27]
- Patient being vulnerable [P34]
- Sexual assault [P41]
- worries they will be exploited, or exploit someone [P57]
- Boundaries, triggering and intrusiveness [P58]
- worries about service user being vulnerable to exploitation [P62]

Support

- Lack of management support [P5]
- Management support and the general perception of the public about people with mental illness dating [P33]
- Other supports and services seeing this as unimportant or inappropriate [P46]
- Management support [P48]
- Lack of support / training around having these conversations [P56]
- nature of service are largest factors [P54]

Training

- I have experienced no training or discussions around this therefore naturally you think it might be out of the scope of your professional boundaries. Something about it does feel that way. [31]
- Lack of support / training around having these conversations [P56]

Societal perception

- Management support and the general perception of the public about people with mental illness dating [P25]
- I would not want to ask someone about finding a relationship if they have not brought it up themselves. This is because I would not want to assume that finding a romantic relationship is something they need or want to do - I don't want to impose societal expectations of relationships on someone, particularly if struggling with their wellbeing. It would need to be worded more broadly, i.e. are you interested in relationships / is this something that's important to you? [P51]

None

- None, it is part of the role to address this aspect of their lives as well in thinking about the person as a whole [P6]

***Question 4, Barriers: ‘Is there anything else you'd like to say about these barriers?’***

Management and policy

- I think the main barrier is management, it is never really spoken about that we need to help the clients find relationships, especially because there are so many people involved that it would need to be discussed with e.g. the care management, social workers, the family. When we’ve had less severe clients in the past who weee definitely capable of relationships and I think would benefit/enjoy it , it was something that was never spoken about or we were trained in /encouraged to help so it was never something we tried to talk about with them. [17]
- I understand their importance but residents do want someone in their life romantically and I want residents to feel happy it would be helpful if there were more clear guidelines on how we can support a client with this [37]
- Everything you do has to be mostly approved or encouraged by them, so even if you have no personal barriers, without management support, it is not something that can be done. [7]

Staff need training

- more training in this regard [41]
- they are very daughting so would i feel staff would like support arund this [33]

Staff may get it wrong

- It could appear as though single staff members are flirting if this conversation was not done effectively [56]

Important issue

- it is very important that this topic is spoken about, this is huge in peoples lives [30]
- It seems a shame because romantic relationships are so important and could be the changing factor in someone's loneliness, suicidality and wellbeing. [31]
- This is a very important area, and I welcome a better understanding of how to navigate these issues and offer a more holistic approach to clients [48]

Client needs to be stable before engaging in relationship

- Need to focus on own mental health and wellbeing and care of a baby. Get to know other people but take things slowly if it works out they will understand. having a baby is one of the most difficult things you'll ever do as a women, and if someone is unwell some awful individuals will take advantage of this. To think about where, when and why your meeting someone new, making friends and family aware so that they know if you do get into any bother. [36]
- Sometimes people, at least in more acute services, might benefit from more stability (e.g. of mpod, of routine, being able to go out) if they are then to find a hood relationship. But this is cery influenced by the fact i wiork in secondary and inpatient services. [51]

***Question 5, Current practice: ‘Please tell us about any ways in which you, or others you work with, try to help those who express a desire for an intimate / romantic relationship.’***

Just having a conversation

- asking a simple question like "What are your thoughts about romantic relationships?" [4]
- Just having a conversation about it and finding out their thoughts. [39]
- conversation, reassurance, practical advice [41]
- Explore whether they have any pre-existing ideas in mind first and take it from there [8]
- I ask about satisfaction with intimate relationships as part of an initial screener/putcome measure. [55]

Discerning client goals

- to ask whether having a relationship is one of their goals [46]
- asking what a person would like in the future or work towards [30]

Exploring relationship desires

- I have chats with my clients about what kind of relationship they want. I try to support them by asking how they like to reach out to significant others i.e. in person/online and discuss safety when dating e.g. keeping to public spaces not sharing personal information etc. more of an advisory role as opposed to engaging them directly. [37]

Discussing barriers

- I guess figure out whats stopping them in the first place or what barriers they have and figure out what we can do to help with those barriers [47]

Discussing social network

- Discussion of social connections [6]
- Optimising mental health can sometimes facilitate this, as can helping people to engage in social activities. But this is an indirect effect of something that is done anyway. [49]

Not done in my service

- This is not something that is done in my practice. [7]
- In 20 years I’ve not witnessed this [40]

Take service user’s lead

- If sex or romantic relationships are specifically raised as an identified issue, this will be explored, formulated and any barriers discussed. Intimate/family relationships is a part of the care plan, however it rarely is a priority to focus on within the time frame. [31]
- Begin to discuss when they bring it up - based around their circumstances there is no guidance [50]

Relationship education

- We try and sensitively explain how consensual relationships work. Remind them how coercive behaviour is wrong and try and guide them on acceptable behaviour [9]
- explore healthy and unhealthy relationships. To think about the whole picture in keeping themselves safe keep talking about it. [36]
- I have chats with my clients about what kind of relationship they want. I try to support them by asking how they like to reach out to significant others i.e. in person/online and discuss safety when dating e.g. keeping to public spaces not sharing personal information etc. more of an advisory role as opposed to engaging them directly. [37]
- We can provide support and encouragement, help people to identify their needs and goals, teach people about communication and relationship skills, and connect people with resources in the community. [45]
- Talking about and having easyread materials available describing qualities of supportive romantic relationships, and dating safety including online. Role playing starting conversations or using drama or discussion to explore feelings around romance and sexuality. Also sadly safeguarding as sometimes this is expressed in the context of abusive relationships. [52]
- Meeting sexual needs in hospital, social skills, online safety [54]

Increasing access to partners

- Direct them to dating sites o encourage their families/carers to support [10]
- look into options of where to find a good match - common interests [27]
- behavioural experiments using dating apps and broadening social opportunities. [31]
- We can provide support and encouragement, help people to identify their needs and goals, teach people about communication and relationship skills, and connect people with resources in the community. [45]

Skills building

- It wouldn’t be about finding a relationship for them but more so about building interpersonal social skills and recognising how they might be vulnerable to exploitation [13]
- Meeting sexual needs in hospital, social skills, online safety [54]

Building self-esteem

- Building up self-esteem [15]

Therapeutic interventions

- Motivational interviewing around meeting others [31],
- Through therapy, clarifying what they want, problrm solving how they might go about it, challenging anxieties or negative thoughts about how it might work out. S [51]
- In LD service we specifically had a sex and relationships group. It comes up in CAMHS but feels less focused on as a part of the work [53]

Showing support for client’s initiative

- i show my support for them going out with friends/to pubs and using dating apps but havn't helped practically [33]
- I have chats with my clients about what kind of relationship they want. I try to support them by asking how they like to reach out to significant others i.e. in person/online and discuss safety when dating e.g. keeping to public spaces not sharing personal information etc. more of an advisory role as opposed to engaging them directly. [37]
- conversation, reassurance, practical advice [41]
- We can provide support and encouragement, help people to identify their needs and goals, teach people about communication and relationship skills, and connect people with resources in the community. [45]

Practical advice

- conversation, reassurance, practical advice [41]
- I have chats with my clients about what kind of relationship they want. I try to support them by asking how they like to reach out to significant others i.e. in person/online and discuss safety when dating e.g. keeping to public spaces not sharing personal information etc. more of an advisory role as opposed to engaging them directly. [37]
- I would say they need to get out of their home to try and meet new people and hope they meet someone that eay [56].

Unsure

- I’m not sure! But I’m only at this service once a week so I may miss a lot of these conversations. I have spoken to clients about past relationships, but not about seeking out future ones since no desire was expressed and there were more pressing matters to address. [38]

Discussion of sexual needs

- Meeting sexual needs in hospital, social skills, online safety [54]

***Question 6, Future suggestions: ‘Are there any other ways you think staff in mental health and social care services could support people in finding a relationship (even if these are not current practice in your workplace)?’***

Attending activities outside the service

- Attending group activities in the community, more opportunities to interact with people outside of the service [6]
- Taking clients to community events e.g. coffee events with other people with disabilities, social dance classes etc. where they can be encouraged to interact with other people in society and potentially form relationships. [7]

Direct support: dating sites

- Maybe helping with dating sites but again i wouldnt know how to support someone to not get taken advantage of here. [33]
- I think it would be good to find dating services (free) that is like speed-dating or friendship making. Or provide this within the service or in the wider organisation so that residents can establish positive relationships [37]

Facilitation within the service

- Social opportunities in services [15]
- Possibly some form of safe dating facilitation - many people who use mental health services have relationships with others who do too. Quite a few fears though about the possibility that resulting relationships might turn out to be problematic in some way.

Accessing social opportunities

- Unsure, it also feels quite strange to offer a specific intervention around it as you can feel like a dating service and it might feel forced. Where as taking a social intervention and allowing any relationships to come naturally seems the more acceptable way of doing it. [31]
- I suppose via signposting to community activities? [38]

Asking about barriers to relationships at assessment

- But explicitly asking about thoughts and barriers to finding romantic relationships should be more prominent in assessment questions and interventions around mental health.. [31]

Group work

- Discussion and group work [32]
- Offer support in the form of groups to discuss what a healthy relationship looks like. Do some scenarios and explore what they would do in that situation. [36]
- Possibly, groups to discuss online dating safety etc. [49]

Open discussion

- Discussing it more openly and not stigmatising [39]
- to give permission to patients to state this as a goal and set out how you might support someone in their socialisation to address this [46]
- Talking about sex and relationships more [54]
- Open conversion. Looking at roles and routines that might support opportunities. We can explore what the emotional, practical and esteem barriers are. There are always options but it takes the service user a lot to over come this. [55]

Psychological preparation

- And by helping to psychologically prepare them for a relationship if needed [38]

Psychoeducation

- offer some eduation about safety in relaitonships for vulnerable peope including udnerstanding their own comfortable boundaries. [33]
- Perhaps psycho Ed on healthy relationships, for some people like LD services maybe learning social skills to develop relationships [53]

Signposting

- Direct them to relevant services [10]
- Training! Being aware of organisations to signpost to that set up safe spaces for more vulnerable people to meet someone. [52]

Skills building (indirect)

- By working on the individual to build skills mentioned above that‚Äôs as far as we should go [13]
- Maybe some skills support around going on dates e.g conversationa dn social interaction [33]
- Support with social skills, ensuring the person is being treated for symptoms that may affect confidence etc, or functioning, addressing sexual side effects or symptoms as well. Improving self-esteem, [48]

Needing more knowledge in this area

- more knowledge around this [27]
- I don't know, and probably should [30]
- Training [40]
- No [41]
- Unsure [56]

Organisational change

- improving policies and access to material to meet needs [54]

Should not be part of MHP role

- I do not think it should be part of my role [9]

**Step 2: Creating overarching themes for each of seven qualitative questions.**

***Question 1, Appropriateness:* ‘*Please explain briefly why you think this is or is not part of your work role.’***

AGREE THEMES

**Encouraging recovery**

Part of holistic care

- Part of holistic care planning [6]
- Part of recovery and happiness [10]
- My patients talk a lot as part of their recovery is ‚”finding love” [23]
- It's a key part of people's lives and recoveries. But does require some delicacy to do well and some evidence on how to do it well would be good too. [26]
- Relationships are an important aspect of overall wellbeing, and health. Whilst we may not be experts at supporting people in forming intimate relationships, or indeed matchmaking, our role should include an interest in all aspects of a person's emotional health - and they should be made to feel comfortable discussing such matters. Just as their physical wellbeing, or wishes regarding employment/education etc are all potentially relevant to their mental state. [30]
- its important for the individual's wellbeing, QoL etc. It can however be difficult to support, and in some instances need to considering safeguarding risks. [27]
- OTs work holistically with all aspects of life [36]
- It is a vital part of identity and role fulfilment [14]
- Relationships are a hugely important part of our identity and wellbeing [P51]
- We consider the social life of the person in many other ways and this is likely to be one of the most important and meaningful aspects of this for the person [P55]
- Relationships are part of well-being. It should be no different to thinking through with someone what they need for other values they have such as employment or exercise. [P61]
- our role is to help a patient holistically [P62]

Good for mental health

- Romantic relationships are key to managing mental health and to manage loneliness [16]
- I feel that romantic relationships and intimacy are a human need and have a huge impact on mental health. [P36]
- Can support mental well-being. Also important to encourage service users to think about risk/disclosure of offences as part of my responsibility to society [P57]
- We know from research that loneliness is integral to physical and mental health, and relationships are relevant to tackling isolation. [P61]
- Depression can be linked with loneliness and a lack of connection with others. Helping someone to try dating again could be really beneficial to their mental health. [33]

**Methods to support client relationships**

Recognising healthy relationships and how to behave in them

- but we should support our service users to recognise healthy relationships, build boundaries and encourage positive social interactions [9]
- To support them to think about what a healthy relationship is and to think about the way in which they meet new people. [19]
- Discussing what a safe and healthy relationship might look like and navigating this [P59]

Self improvement support

- I would support with self-improvement goals to help the chances of finding a relationship e.g. healthy living, physical fitness, employment, hobbies [13]

Skills building

- however, helping them to develop appropriate skills would help to build therapeutic relationship and may result in better mental health outcomes for them. [3]
- Agree that supporting with general social skills and areas that support development in this area is part of the role. [P59]

Help to access potential partners

- We can help people attend groups etc or meet new people but we can‚Äôt really promote anything that isn‚Äôt regulated. Would be a challenge to help them find this relationship [38]

**General importance**

Therapeutic relevance

- Patients are concerned regarding body image and struggle with intimacy [17]
- I think it should be more because it is a strong motivator and some patients do not have experience of developing relationships of any kind [32]
- As a psychologist, I believe we have some capacity to help people to consider their needs and roles in relationships. [21]
- I believe this is important. Because many service users can struggle to form relationships in general, and having some support regarding intimate relationship would be useful for these users. [P58]

Service users want support

- I think people want this but do not express it as they don’t feel it is within our role [18]

Service user’s desire

- Having a meaningful relationship with someone is many peoples goal. it is very much my role to help/support/advise in ways I can [15]
- this is a pressing concern and need in the service users I support [24]

**Caveats**

Needing limits

- I think as long as you keep it within certain boundaries, why shouldn’t you support them? [22]
- Whatever the occupational barriers might be, i would explore goals and ways of overcoming them. There would be limits however. [37]
- Whilst I don’t think we should be advising someone if they should/shouldnt be in a relationship. Sex and relationships are a significant part of identity and if it’s something that someone wants to explore, wants to think about, patterns, barriers etc, then it’s of course part of our job. [P56]

Only appropriate if mental health related

- If they are struggling to maintain or find a relationship based on MH difficulties then i understand that our role can help them with self-esteem or any social anxiety but i think its only our work role if its MH related, not just because someone without MH cant find a relationship. Not sure how we are suppose to help there [29]
- I would see supporting someone to develop their skills and knowledge around navigating romantic relationships as a part of my role if important to the person. Particularly if mental health difficulties or learning difficulties or neurodivergence was a barrier to this.. [34]

DISAGREE THEMES

**Moral and ethical issues**

Unethical

- I feel like it would be unethical to help them find a relationship [3]
- psychologists and most other professionals are not trained in discussing or particularly advising around seeking/developing romantic relationships, and so it may not always be appropriate to do as it would likely be informed by subjective opinion, and could be harmful. [P51]

Inappropriate

- I'm not sure how appropriate helping someone form an intimate relationship is? [12]
- It feels slightly intrusive to become involved in someone's romantic life; a breach of a professional boundary. [31]

Safeguarding risks

- It can however be difficult to support, and in some instances need to considering safeguarding risks. [27]
- There are also risks to be considered, for example of people ending up in abusive relationships/one with very unequal power dynamics. [26]

Professional responsibility

- I would support with self-improvement goals to help the chances of finding a relationship e.g. healthy living, physical fitness, employment, hobbies, but not with actually finding a relationship as there is a concern about professional responsibility if something goes wrong

Abusive clients

- Many of the clients have history of being perpetrators of DV. [7]

**Not feasible in my job role**

Work role is closely delineated

- My work role involves logging into an app at the start of the shift, and ticking off the tasks that are listed e.g. prepare food, so the work is mostly set out for us. We do help assist with emotional support and distress, but no tasks are really geared towards building relationships. [5]
- ). I also do outreach as part of my job with an autistic male who is more high functioning, and has said once that he seeks a relationship. My role when I started was to encourage him to try new activities in London and discuss any emotional difficulties if they arise (such as struggling with depression), and I think that is the role I feel responsible for as opposed to helping them find a partner. [5]

Can’t be matchmakers

- We cannot act as matchmakers [9]
- It‚Äôs relevant to mental health, and somewhat relevant in terms of values and behaviour, but actually practically finding somebody feels a little beyond our scope [35]

Relying on other professionals

- tend to rely on support workers to help them socialise but can suggest this as part of the care plan [28]
- However, perhaps social workers / OTs within my team could run groups on safety / appropriate when online dating etc. which would be in keeping with this aim. [31]

**Relationships are irrelevant for my clients**

Desire and capability

- Additionally, the clients have severe mental/physical disabilities and so it is either not something they would desire (e.g. not mentally capable of), or they are too severe to interact with the public/other society members e.g. prone to anger and attacking (e.g. not socially capable of) [5]

Not something service users bring up

- I think that is also how they view me so he wouldn‚Äôt necessarily go to me for that (possibly because they don‚Äôt deem as appropriate) unless I approached the topic. [5]

NEITHER AGREE NOR DISAGREE THEMES

Depends on their level of care: high support not priority

- It depends on the level of care they require, in the more independent housing I would say I would be happy to discuss this but in high support this is not our priority [4]
- Nature of the service/ acute emergency assessments but I would strongly agree for treatment/ community services. who work with people longer term. [P54]

Undecided

- I'm undecided I think it could be good for residents to find meaningful relationships but I wonder if it crosses a boundary to support them into those relationships [20]

***Question 2, Barriers:* ‘*How much do the following factors form barriers to “finding a relationship” conversations? - Other barrier (if applicable, please specify)’***

**A relationship is not appropriate for service user**

Relationship being detrimental to the service user

- it is probable that a relationship may present another stressor to the service user's life (moderate amount) [P2]

Service user not capable of relationship

- A view that the individual may not be socially capable of a relationship e.g. if they display traits of aggression [P5]

Service user may put their partner at risk

- The clients who have asked are male and have a history of perpetrators of domestic violence [P7]
- Might be a DV perpetrator themselves so this comes with risks [P25]

Service user not ready for a relationship

- just had a baby, might not be ready yet. [P30]

**Worrying about not being capable**

Liability

- Worry that if it goes wrong will the resident blame me? [P31]

Making things worse

- If a person has a severe learning disability and has always been single, conversations about relationships (if handled incorrectly)may come across as condescending or as if you are making fun of them [P13]
- also concerns that the discussion (where initiated by clinician) stigmatise their single status) [P40]

Being ill equipped

- Also feel inequipped like it would be opening a tin of worms [P44]

Other people are better suited to this work

- I think I'm not necessarily the best person as a consultant psychiatrist carrying out relatively formal reviews - conversations better initiated by people like care coordinators or support workers in a relatively informal setting. [P38]

**Organisational factors**

Not part of routine practice

- Relationship goals not being part of routine assessment [P46]

Colleague hesitation

- Colleagues opinions of whether it's our role [P49]

Not appropriate context

- starting up relationship problems in a therapeutic session [P2]

Systemic deprioritisation

- Consideration of relationships fits a social/biopsychosocial model of mental health, but many services and service users are still within an 'illness' model where holistic conversations re: wider relationships may not be a) trained, b) seen as appropriate [P51]

**Communication issues**

Needing therapeutic alliance first

- People not wanting to discuss this until they have built a trusting relationship with the professional [P46]

Service user not asking for support

- The person who we support being able to properly communicate their desire for a relationship even if it is something they might want [P13]

**External factors**

Families being overprotective

- If the family are active in the person we supports lives, they could be overprotective and not want their child to have a relationship e.g. they feel they are too vulnerable or would not want staff to be involved in that way [P13]
- If the family are heavily involved, they can often be overprotective and not want their child to be dating [P5]

Related barriers

- Other support around the person such as family or services being opposed [P46]
- service users' previous relationship problems [P2]

***Question 3, Barriers: ‘Which of these barriers do you think is the most significant?’***

**Inappropriate for clinician**

Inappropriate in my work role

- feeling it is inappropriate in my work role [P2]
- Worries that is inappropriate and intrusive [P4]
- Concerns re: relevance/appropriateness - not personally, but at service level and service user level. [P51]

Breaking boundaries

- Worries about it breaking professional boundaries [P9]
- Ensuring the support remains boundaries and the service user takes the lead if this is what they wish to do [P10]
- professional boundaries [P17]
- Professional boundaries, [P19]
- The barrier around professional boundaries [P31]
- crossing professional lines [P35]
- Boundaries [P51]
- Boundaries, triggering and intrusiveness [P58]

Intrusive

- Worries that is inappropriate and intrusive [P4]
- worries it might seem too intrusive [P41]
- Being intrusive to the service user. Also, a make worker asking a female might be viewed dimly or inappropriate. [P49]
- Boundaries, triggering and intrusiveness [P58]

Gender issues between client and clinician

- Being intrusive to the service user. Also, a make worker asking a female might be viewed dimly or inappropriate. [P49]
- Nature of the service and being a male member of staff I would not feel comfortable discussing with a female patient and would also be concerned that this would make them uncomfortable. [P54]
- I think gender of staff member [P54]

Relationships not prioritised

- Prioritising other issues [P45]

**Clinician inability**

Lack of training

- Lack of training [P32]
- Lack of management training [P13]
- I have experienced no training or discussions around this therefore naturally you think it might be out of the scope of your professional boundaries. Something about it does feel that way. [P25]
- Lack of training around conversations and how these can be appropriate [P36]
- Lack of training perhaps [P42]
- Lack of training [P47]

Hard to identify clients

- Hard to identify just one and varies from person to person [P38]

Lack of time and resources

- Time [P8]
- Time and appropriate services [P12]
- Time, and feeling unable to help. [P43]
- Time constraints are a factor [P27]

Low clinician confidence

- voulnerability. I'm and experienced nurse although low confidence with this [30]
- not knowing how to support someone to seek a relationship and worry about their vulnerability [P24]
- Time, and feeling unable to help. [P43]
- Not feeling able to help [P57]
- Not being able to do anything to help if they want a relationship [P59]

**Client factors**

Client’s history

- Client’s history of domestic violence [P7]
- Sexual assault [P41]

Client context

- Either there pregnant or have a baby up to the age of 2. May need support for this before getting into a new relationship. [P30]

Vulnerable patients

- vulnerable patients [P19]
- voulnerability. I'm and experienced nurse although low confidence with this [P24]
- not knowing how to support someone to seek a relationship and worry about their vulnerability [P27]
- Patient being vulnerable [P34]
- Sexual assault [P41]
- worries they will be exploited, or exploit someone [P57]
- Boundaries, triggering and intrusiveness [P58]
- worries about service user being vulnerable to exploitation [P62]

**Organisational factors**

Support

- Lack of management support [P5]
- Management support and the general perception of the public about people with mental illness dating [P33]
- Other supports and services seeing this as unimportant or inappropriate [P46]
- Management support [P48]
- Lack of support / training around having these conversations [P56]
- nature of service are largest factors [P54]

Training

- I have experienced no training or discussions around this therefore naturally you think it might be out of the scope of your professional boundaries. Something about it does feel that way. [31]
- Lack of support / training around having these conversations [P56]

**Societal perception**

- Management support and the general perception of the public about people with mental illness dating [P25]
- I would not want to ask someone about finding a relationship if they have not brought it up themselves. This is because I would not want to assume that finding a romantic relationship is something they need or want to do - I don't want to impose societal expectations of relationships on someone, particularly if struggling with their wellbeing. It would need to be worded more broadly, i.e. are you interested in relationships / is this something that's important to you? [P51]

**None**

- None, it is part of the role to address this aspect of their lives as well in thinking about the person as a whole [P6]

***Question 4, Barriers: ‘Is there anything else you'd like to say about these barriers?’***

**More training and support needed**

Management and policy

- I think the main barrier is management, it is never really spoken about that we need to help the clients find relationships, especially because there are so many people involved that it would need to be discussed with e.g. the care management, social workers, the family. When we’ve had less severe clients in the past who weee definitely capable of relationships and I think would benefit/enjoy it , it was something that was never spoken about or we were trained in /encouraged to help so it was never something we tried to talk about with them. [17]
- I understand their importance but residents do want someone in their life romantically and I want residents to feel happy it would be helpful if there were more clear guidelines on how we can support a client with this [37]
- Everything you do has to be mostly approved or encouraged by them, so even if you have no personal barriers, without management support, it is not something that can be done. [7]

Staff need training

- more training in this regard [41]
- they are very daughting so would i feel staff would like support arund this [33]

**Staff may get it wrong**

- It could appear as though single staff members are flirting if this conversation was not done effectively [56]

**Important issue**

- it is very important that this topic is spoken about, this is huge in peoples lives [30]
- It seems a shame because romantic relationships are so important and could be the changing factor in someone's loneliness, suicidality and wellbeing. [31]
- This is a very important area, and I welcome a better understanding of how to navigate these issues and offer a more holistic approach to clients [48]

**Client needs to be stable before engaging in relationship**

- Need to focus on own mental health and wellbeing and care of a baby. Get to know other people but take things slowly if it works out they will understand. having a baby is one of the most difficult things you'll ever do as a women, and if someone is unwell some awful individuals will take advantage of this. To think about where, when and why your meeting someone new, making friends and family aware so that they know if you do get into any bother. [36]
- Sometimes people, at least in more acute services, might benefit from more stability (e.g. of mpod, of routine, being able to go out) if they are then to find a hood relationship. But this is cery influenced by the fact i wiork in secondary and inpatient services. [51]

***Question 5, Current practice: ‘Please tell us about any ways in which you, or others you work with, try to help those who express a desire for an intimate / romantic relationship.’***

**Discussions with service user**

Just having a conversation

- asking a simple question like "What are your thoughts about romantic relationships?" [P2]
- Just having a conversation about it and finding out their thoughts. [P33]
- conversation, reassurance, practical advice [P35]
- Explore whether they have any pre-existing ideas in mind first and take it from there [P6]
- I ask about satisfaction with intimate relationships as part of an initial screener/putcome measure. [P49]
- conversations re: navigating sex and consent. [P51]
- Exploring ideas around relationships, sex, where they come from. [P56]

Discerning client goals

- to ask whether having a relationship is one of their goals [P40]
- asking what a person would like in the future or work towards [P24]
- I have chats with my clients about what kind of relationship they want. I try to support them by asking how they like to reach out to significant others i.e. in person/online and discuss safety when dating e.g. keeping to public spaces not sharing personal information etc. more of an advisory role as opposed to engaging them directly. [P31]

Discussing barriers

- I guess figure out whats stopping them in the first place or what barriers they have and figure out what we can do to help with those barriers [P41]

Discussing social network

- Discussion of social connections [P4]
- Optimising mental health can sometimes facilitate this, as can helping people to engage in social activities. But this is an indirect effect of something that is done anyway. [P43]

Discussion of sexual needs

- Meeting sexual needs in hospital, social skills, online safety [P48]

**Teaching and advice**

Relationship education

- We try and sensitively explain how consensual relationships work. Remind them how coercive behaviour is wrong and try and guide them on acceptable behaviour [P7]
- explore healthy and unhealthy relationships. To think about the whole picture in keeping themselves safe keep talking about it. [P30]
- I have chats with my clients about what kind of relationship they want. I try to support them by asking how they like to reach out to significant others i.e. in person/online and discuss safety when dating e.g. keeping to public spaces not sharing personal information etc. more of an advisory role as opposed to engaging them directly. [P31]
- We can provide support and encouragement, help people to identify their needs and goals, teach people about communication and relationship skills, and connect people with resources in the community. [P39]
- Talking about and having easy read materials available describing qualities of supportive romantic relationships, and dating safety including online. Role playing starting conversations or using drama or discussion to explore feelings around romance and sexuality. Also sadly safeguarding as sometimes this is expressed in the context of abusive relationships. [P46]
- Meeting sexual needs in hospital, social skills, online safety [P48]
- Discuss safety and healthy relationships and what they look like [P59]

Practical advice

- conversation, reassurance, practical advice [P35]
- I have chats with my clients about what kind of relationship they want. I try to support them by asking how they like to reach out to significant others i.e. in person/online and discuss safety when dating e.g. keeping to public spaces not sharing personal information etc. more of an advisory role as opposed to engaging them directly. [P31]
- I would say they need to get out of their home to try and meet new people and hope they meet someone that way [P50].
- Recommendations re: dating apps, approaches to dating [P51]

**Active interventions**

Increasing access to partners

- Direct them to dating sites o encourage their families/carers to support [P8]
- look into options of where to find a good match - common interests [P21]
- behavioural experiments using dating apps and broadening social opportunities. [P25]
- We can provide support and encouragement, help people to identify their needs and goals, teach people about communication and relationship skills, and connect people with resources in the community. [P39]
- Support to meet people online, discuss progress [P57]

Skills building

- It wouldn’t be about finding a relationship for them but more so about building interpersonal social skills and recognising how they might be vulnerable to exploitation [P10]
- Meeting sexual needs in hospital, social skills, online safety [P48]

Building self-esteem

- Building up self-esteem [P11]
- building self confidence [P51]

Therapeutic interventions

- Motivational interviewing around meeting others [P25]
- Through therapy, clarifying what they want, problrm solving how they might go about it, challenging anxieties or negative thoughts about how it might work out. S [P45]
- In LD service we specifically had a sex and relationships group. It comes up in CAMHS but feels less focused on as a part of the work [P47]

**None known**

Unsure

- I’m not sure! But I’m only at this service once a week so I may miss a lot of these conversations. I have spoken to clients about past relationships, but not about seeking out future ones since no desire was expressed and there were more pressing matters to address. [P32]

Not done in my service

- This is not something that is done in my practice. [P5]
- In 20 years I’ve not witnessed this [P34]
- None [P58]

**Taking service user’s lead**

- If sex or romantic relationships are specifically raised as an identified issue, this will be explored, formulated and any barriers discussed. Intimate/family relationships is a part of the care plan, however it rarely is a priority to focus on within the time frame. [P25]
- Begin to discuss when they bring it up - based around their circumstances there is no guidance [P44]

***Question 6, Future suggestions: ‘Are there any other ways you think staff in mental health and social care services could support people in finding a relationship (even if these are not current practice in your workplace)?’***

**Opportunities for socialising**

Attending social activities outside the service

- Attending group activities in the community, more opportunities to interact with people outside of the service [P4]
- Taking clients to community events e.g. coffee events with other people with disabilities, social dance classes etc. where they can be encouraged to interact with other people in society and potentially form relationships. [P5]
- Unsure, it also feels quite strange to offer a specific intervention around it as you can feel like a dating service and it might feel forced. Where as taking a social intervention and allowing any relationships to come naturally seems the more acceptable way of doing it. [P25]

Facilitation within the service

- Social opportunities in services [P11]
- Possibly some form of safe dating facilitation - many people who use mental health services have relationships with others who do too. Quite a few fears though about the possibility that resulting relationships might turn out to be problematic in some way [P38].

Finding dating services

- Maybe helping with dating sites but again i wouldnt know how to support someone to not get taken advantage of here. [P27]
- I think it would be good to find dating services (free) that is like speed-dating or friendship making. Or provide this within the service or in the wider organisation so that residents can establish positive relationships [P31]

Signposting

- I suppose via signposting to community activities? [P32]

**More discussion about relationships in service**

Asking about barriers to relationships at assessment

- But explicitly asking about thoughts and barriers to finding romantic relationships should be more prominent in assessment questions and interventions around mental health.. [P25]

Group work

- Discussion and group work [P26]
- Offer support in the form of groups to discuss what a healthy relationship looks like. Do some scenarios and explore what they would do in that situation. [P30]
- Possibly, groups to discuss online dating safety etc. [P43]

Open discussion

- Discussing it more openly and not stigmatising [P33]
- to give permission to patients to state this as a goal and set out how you might support someone in their socialisation to address this [P40]
- Talking about sex and relationships more [P48]
- Open conversion. Looking at roles and routines that might support opportunities. We can explore what the emotional, practical and esteem barriers are. There are always options but it takes the service user a lot to over come this. [P49]

Psychoeducation

- offer some eduation about safety in relaitonships for vulnerable peope including udnerstanding their own comfortable boundaries. [P27]
- Perhaps psycho Ed on healthy relationships, for some people like LD services maybe learning social skills to develop relationships [P47]
- And by helping to psychologically prepare them for a relationship if needed [P32]

**Indirect support**

Signposting to services

- Direct them to relevant services [P8]
- Training! Being aware of organisations to signpost to that set up safe spaces for more vulnerable people to meet someone. [P46]

Skills building

- By working on the individual to build skills mentioned above that’s as far as we should go [P10]
- Maybe some skills support around going on dates e.g conversationa dn social interaction [P27]
- Support with social skills, ensuring the person is being treated for symptoms that may affect confidence etc, or functioning, addressing sexual side effects or symptoms as well. Improving self-esteem, [P42]

**Systemic change**

Needing more knowledge in this area

- more knowledge around this [P21]
- I don't know, and probably should [P24]
- Training [P34]
- No [P35]
- Unsure [P50]

Organisational change

- improving policies and access to material to meet needs [P48]

**Should not be part of MHP role**

- I do not think it should be part of my role [P7]

**Step 3: Merging seven analyses into four.**

***Merge 1: Barriers***

**Provider factors**

Provider not feeling able to help

- Worry that if it goes wrong will the resident blame me? [P31]
- If a person has a severe learning disability and has always been single, conversations about relationships (if handled incorrectly)may come across as condescending or as if you are making fun of them [P13]
- also concerns that the discussion (where initiated by clinician) stigmatise their single status) [P40]
- Also feel inequipped like it would be opening a tin of worms [P44]
- I think I'm not necessarily the best person as a consultant psychiatrist carrying out relatively formal reviews - conversations better initiated by people like care coordinators or support workers in a relatively informal setting. [P38]
- voulnerability. I'm and experienced nurse although low confidence with this [P30]
- not knowing how to support someone to seek a relationship and worry about their vulnerability [P24]
- Time, and feeling unable to help. [P43]
- Not feeling able to help [P57]
- Not being able to do anything to help if they want a relationship [P59]
- Hard to identify just one and varies from person to person [P38]

Inappropriate for the provider

- feeling it is inappropriate in my work role [P2]
- Worries that is inappropriate and intrusive [P4]
- Concerns re: relevance/appropriateness - not personally, but at service level and service user level. [P51]
- Worries about it breaking professional boundaries [P9]
- Ensuring the support remains boundaries and the service user takes the lead if this is what they wish to do [P10]
- professional boundaries [P17]
- Professional boundaries, [P19]
- The barrier around professional boundaries [P31]
- crossing professional lines [P35]
- Boundaries [P51]
- Boundaries, triggering and intrusiveness [P58]
- Worries that is inappropriate and intrusive [P4]
- worries it might seem too intrusive [P41]
- Being intrusive to the service user. Also, a make worker asking a female might be viewed dimly or inappropriate. [P49]
- Boundaries, triggering and intrusiveness [P58]
- Being intrusive to the service user. Also, a make worker asking a female might be viewed dimly or inappropriate. [P49]
- Nature of the service and being a male member of staff I would not feel comfortable discussing with a female patient and would also be concerned that this would make them uncomfortable. [P54]
- I think gender of staff member [P54]
- Prioritising other issues [P45]

Communication issues

- The person who we support being able to properly communicate their desire for a relationship even if it is something they might want [P13]
- People not wanting to discuss this until they have built a trusting relationship with the professional [P46]

**Service user factors**

Relationships are inappropriate for the service user

- just had a baby, might not be ready yet. [P30]
- The clients who have asked are male and have a history of perpetrators of domestic violence [P7]
- Might be a DV perpetrator themselves so this comes with risks [P25]
- A view that the individual may not be socially capable of a relationship e.g. if they display traits of aggression [P5]
- it is probable that a relationship may present another stressor to the service user's life (moderate amount) [P2]
- Client’s history of domestic violence [P7]
- Sexual assault [P41]
- Either there pregnant or have a baby up to the age of 2. May need support for this before getting into a new relationship. [P30]
- vulnerable patients [P19]
- voulnerability. I'm and experienced nurse although low confidence with this [P24]
- not knowing how to support someone to seek a relationship and worry about their vulnerability [P27]
- Patient being vulnerable [P34]
- Sexual assault [P41]
- worries they will be exploited, or exploit someone [P57]
- Boundaries, triggering and intrusiveness [P58]
- worries about service user being vulnerable to exploitation [P62]
- service users' previous relationship problems [P2]

Service user must be stable

- Need to focus on own mental health and wellbeing and care of a baby. Get to know other people but take things slowly if it works out they will understand. having a baby is one of the most difficult things you'll ever do as a women, and if someone is unwell some awful individuals will take advantage of this. To think about where, when and why your meeting someone new, making friends and family aware so that they know if you do get into any bother. [36]
- Sometimes people, at least in more acute services, might benefit from more stability (e.g. of mpod, of routine, being able to go out) if they are then to find a hood relationship. But this is cery influenced by the fact i wiork in secondary and inpatient services. [51]

**Organisational factors**

Lack of support

- Colleagues opinions of whether it's our role [P49]
- Consideration of relationships fits a social/biopsychosocial model of mental health, but many services and service users are still within an 'illness' model where holistic conversations re: wider relationships may not be a) trained, b) seen as appropriate [P51]
- Lack of management support [P5]
- Management support and the general perception of the public about people with mental illness dating [P33]
- Other supports and services seeing this as unimportant or inappropriate [P46]
- Management support [P48]
- Lack of support / training around having these conversations [P56]
- nature of service are largest factors [P54]
- I think the main barrier is management, it is never really spoken about that we need to help the clients find relationships, especially because there are so many people involved that it would need to be discussed with e.g. the care management, social workers, the family. When we’ve had less severe clients in the past who weee definitely capable of relationships and I think would benefit/enjoy it , it was something that was never spoken about or we were trained in /encouraged to help so it was never something we tried to talk about with them. [17]
- Everything you do has to be mostly approved or encouraged by them, so even if you have no personal barriers, without management support, it is not something that can be done. [7]

Lack of training

- I have experienced no training or discussions around this therefore naturally you think it might be out of the scope of your professional boundaries. Something about it does feel that way. [31]
- Lack of support / training around having these conversations [P56]
- Lack of training [P32]
- Lack of management training [P13]
- Lack of training around conversations and how these can be appropriate [P36]
- Lack of training perhaps [P42]
- Lack of training [P47]
- more training in this regard [41]
- they are very daughting so would i feel staff would like support arund this [33]
- It could appear as though single staff members are flirting if this conversation was not done effectively [56]

Lack of policy

- Relationship goals not being part of routine assessment [P46]
- starting up relationship problems in a therapeutic session [P2]
- I understand their importance but residents do want someone in their life romantically and I want residents to feel happy it would be helpful if there were more clear guidelines on how we can support a client with this [37]

Lack of time and resources

- Time [P8]
- Time and appropriate services [P12]
- Time, and feeling unable to help. [P43]
- Time constraints are a factor [P27]
- Even though this is important, it is difficult to address these issues in the time slot given to me to see the service user. I also do not have access to resources or skills to be able to help someone like that. [8]
- Also, there is barely enough time to do the core aspects of my job, so in my opinion there is very unlikely to be resource for this. [31]
- services are usually very limited in the type of therapeutic intervention we are 'allowed' to offer due to funding and commissioning, and focused on particular mental-health related outcomes (which while linked to relationships, are not usually defined as such). [P51]

**External factors**

Families being overprotective

- If the family are active in the person we supports lives, they could be overprotective and not want their child to have a relationship e.g. they feel they are too vulnerable or would not want staff to be involved in that way [P13]
- If the family are heavily involved, they can often be overprotective and not want their child to be dating [P5]

Societal perception

- Other support around the person such as family or services being opposed [P46]
- Management support and the general perception of the public about people with mental illness dating [P25]
- I would not want to ask someone about finding a relationship if they have not brought it up themselves. This is because I would not want to assume that finding a romantic relationship is something they need or want to do - I don't want to impose societal expectations of relationships on someone, particularly if struggling with their wellbeing. It would need to be worded more broadly, i.e. are you interested in relationships / is this something that's important to you? [P51]

**None**

- None, it is part of the role to address this aspect of their lives as well in thinking about the person as a whole [P6]

***Merge 2: Current practice and suggestions***

**Current practice**

Preparing the service user for the dating world

- It wouldn’t be about finding a relationship for them but more so about building interpersonal social skills and recognising how they might be vulnerable to exploitation [P10]
- Meeting sexual needs in hospital, social skills, online safety [P48]
- Building up self-esteem [P11]
- building self confidence [P51]
- Motivational interviewing around meeting others [P25]
- Through therapy, clarifying what they want, problrm solving how they might go about it, challenging anxieties or negative thoughts about how it might work out. S [P45]
- In LD service we specifically had a sex and relationships group. It comes up in CAMHS but feels less focused on as a part of the work [P47]
- We try and sensitively explain how consensual relationships work. Remind them how coercive behaviour is wrong and try and guide them on acceptable behaviour [P7]
- explore healthy and unhealthy relationships. To think about the whole picture in keeping themselves safe keep talking about it. [P30]
- I have chats with my clients about what kind of relationship they want. I try to support them by asking how they like to reach out to significant others i.e. in person/online and discuss safety when dating e.g. keeping to public spaces not sharing personal information etc. more of an advisory role as opposed to engaging them directly. [P31]
- We can provide support and encouragement, help people to identify their needs and goals, teach people about communication and relationship skills, and connect people with resources in the community. [P39]
- Talking about and having easy read materials available describing qualities of supportive romantic relationships, and dating safety including online. Role playing starting conversations or using drama or discussion to explore feelings around romance and sexuality. Also sadly safeguarding as sometimes this is expressed in the context of abusive relationships. [P46]
- Meeting sexual needs in hospital, social skills, online safety [P48]
- Discuss safety and healthy relationships and what they look like [P59]
- conversation, reassurance, practical advice [P35]
- I have chats with my clients about what kind of relationship they want. I try to support them by asking how they like to reach out to significant others i.e. in person/online and discuss safety when dating e.g. keeping to public spaces not sharing personal information etc. more of an advisory role as opposed to engaging them directly. [P31]
- I would say they need to get out of their home to try and meet new people and hope they meet someone that way [P50].
- Recommendations re: dating apps, approaches to dating [P51]

Discussions with service user about relationships

- asking a simple question like "What are your thoughts about romantic relationships?" [P2]
- Just having a conversation about it and finding out their thoughts. [P33]
- conversation, reassurance, practical advice [P35]
- Explore whether they have any pre-existing ideas in mind first and take it from there [P6]
- I ask about satisfaction with intimate relationships as part of an initial screener/putcome measure. [P49]
- conversations re: navigating sex and consent. [P51]
- Exploring ideas around relationships, sex, where they come from. [P56]
- to ask whether having a relationship is one of their goals [P40]
- asking what a person would like in the future or work towards [P24]
- I have chats with my clients about what kind of relationship they want. I try to support them by asking how they like to reach out to significant others i.e. in person/online and discuss safety when dating e.g. keeping to public spaces not sharing personal information etc. more of an advisory role as opposed to engaging them directly. [P31]
- I guess figure out whats stopping them in the first place or what barriers they have and figure out what we can do to help with those barriers [P41]
- Discussion of social connections [P4]
- Optimising mental health can sometimes facilitate this, as can helping people to engage in social activities. But this is an indirect effect of something that is done anyway. [P43]
- Meeting sexual needs in hospital, social skills, online safety [P48]

Increasing access to partners

- Direct them to dating sites o encourage their families/carers to support [P8]
- look into options of where to find a good match - common interests [P21]
- behavioural experiments using dating apps and broadening social opportunities. [P25]
- We can provide support and encouragement, help people to identify their needs and goals, teach people about communication and relationship skills, and connect people with resources in the community. [P39]
- Support to meet people online, discuss progress [P57]

**Suggestions for future practice**

Psycho-education and skills work

- Discussion and group work [P26]
- Offer support in the form of groups to discuss what a healthy relationship looks like. Do some scenarios and explore what they would do in that situation. [P30]
- Possibly, groups to discuss online dating safety etc. [P43]
- offer some eduation about safety in relaitonships for vulnerable peope including udnerstanding their own comfortable boundaries. [P27]
- Perhaps psycho Ed on healthy relationships, for some people like LD services maybe learning social skills to develop relationships [P47]
- And by helping to psychologically prepare them for a relationship if needed [P32]
- By working on the individual to build skills mentioned above that’s as far as we should go [P10]
- Maybe some skills support around going on dates e.g conversationa dn social interaction [P27]
- Support with social skills, ensuring the person is being treated for symptoms that may affect confidence etc, or functioning, addressing sexual side effects or symptoms as well. Improving self-esteem, [P42]

Open discussion about relationships in service

- But explicitly asking about thoughts and barriers to finding romantic relationships should be more prominent in assessment questions and interventions around mental health.. [P25]
- Discussing it more openly and not stigmatising [P33]
- to give permission to patients to state this as a goal and set out how you might support someone in their socialisation to address this [P40]
- Talking about sex and relationships more [P48]
- Open conversion. Looking at roles and routines that might support opportunities. We can explore what the emotional, practical and esteem barriers are. There are always options but it takes the service user a lot to over come this. [P49]

Increasing access to partners

- Attending group activities in the community, more opportunities to interact with people outside of the service [P4]
- Taking clients to community events e.g. coffee events with other people with disabilities, social dance classes etc. where they can be encouraged to interact with other people in society and potentially form relationships. [P5]
- Unsure, it also feels quite strange to offer a specific intervention around it as you can feel like a dating service and it might feel forced. Where as taking a social intervention and allowing any relationships to come naturally seems the more acceptable way of doing it. [P25]
- Social opportunities in services [P11]
- Possibly some form of safe dating facilitation - many people who use mental health services have relationships with others who do too. Quite a few fears though about the possibility that resulting relationships might turn out to be problematic in some way [P38].
- Maybe helping with dating sites but again i wouldnt know how to support someone to not get taken advantage of here. [P27]
- I think it would be good to find dating services (free) that is like speed-dating or friendship making. Or provide this within the service or in the wider organisation so that residents can establish positive relationships [P31]

Systemic change

- more knowledge around this [P21]
- I don't know, and probably should [P24]
- Training [P34]
- No [P35]
- Unsure [P50]
- improving policies and access to material to meet needs [P48]

Signposting

- Direct them to relevant services [P8]
- Training! Being aware of organisations to signpost to that set up safe spaces for more vulnerable people to meet someone. [P46]
- I suppose via signposting to community activities? [P32]
