# Supplementary material for: ‘Finding a relationship’: Conversations between mental health and social care staff, and service users about service users’ romantic relationships
Source: PLOS Ment Health. 2025 May 8;2(5):e0000184. doi: 10.1371/journal.pmen.0000184 (PMC12798258; doi:10.1371/journal.pmen.0000184)
Supplement: S3 Text — (DOCX) [file pmen.0000184.s003.docx]

**S3 Appendix**

**Collated quantitative results tables**

**Table 1. Participant characteristics.**

| **Variables**  **(Total *N* responses)** |  | *n* (%) |
| --- | --- | --- |
| Gender |  |  |
| (*N* = 63) | Female | 54 (85.7) |
|  | Male | 9 (14.3) |
| Age |  |  |
| (*N* = 63) | 18-25 | 12 (19.1) |
|  | 26-35 | 24 (38.1) |
|  | 36-45 | 15 (23.8) |
|  | 46-55 | 8 (12.7) |
|  | 56-65 | 4 (6.4) |
|  | 65+ | 0 (0) |
| Ethnicity |  |  |
| (*N* = 63) | White | 49 (77.8) |
|  | Asian or Asian British | 6 (9.5) |
|  | Mixed or multiple ethnic groups | 5 (7.9) |
|  | Black, black British, Caribbean or African | 2 (3.2) |
| Religion |  |  |
| (*N* = 63) | No religion | 44 (69.8) |
|  | Christianity | 10 (15.9) |
|  | Buddhism | 3 (4.8) |
|  | Islam | 3 (4.8) |
|  | Hinduism | 1 (1.6) |
|  | Other | 1 (1.6) |
|  | Judaism | 0 (0) |
|  | Sikhism | 0 (0) |
| Years in mental health services |  |  |
| (*N* = 60) | Less than 2 years | 11 (18.3) |
|  | 2-5 years | 19 (31.7) |
|  | 6-10 years | 14 (23.3) |
|  | More than 10 years | 16 (26.7) |
|  |  |  |
| Professional group |  |  |
| (*N* = 60) | Psychologist | 16 (26.7) |
|  | Occupational therapist | 9 (15.0) |
|  | Psychiatrist | 9 (15.0) |
|  | Support worker | 9 (15.0) |
|  | Nurse | 6 (10.0) |
|  | Peer support worker | 3 (5.0) |
|  | Social worker | 3 (5.0) |
|  | Counsellor / therapist | 2 (3.3) |
|  | Other | 2 (3.3) |
| Sector |  |  |
| (*N* = 60) | NHS | 46 (76.7) |
|  | Independent sector | 7 (11.7) |
|  | Voluntary sector | 5 (8.3) |
|  | Local authority | 0 (0) |
| Service type |  |  |
| (*N* = 60) | NHS community mental health team | 33 (55.0) |
|  | Supported accommodation | 10 (16.7) |
|  | Inpatient | 9 (15) |
|  | Other | 5 (8.5) |

**Table 2. Ratings of agreement as to the appropriateness of ‘finding a relationship’ conversations.**

| Agreement as to appropriateness of ‘finding a relationship’ conversations | ***n* (%)**  (total N = 50) |
| --- | --- |
| Strongly agree | 10 (20.0) |
| Somewhat agree | 25 (50.0) |
| Somewhat disagree | 11 (22.0) |
| Strongly disagree | 4 (8.0) |

**Table 4. Participant ratings of the importance of potential barriers to helping service users find intimate relationships.**

|  | Importance rating | |  | |  | |  | |
| --- | --- | --- | --- | --- | --- | --- | --- | --- |
| Barrier | Not at all  *n* (%) | A little  *n* (%) | | A moderate amount  *n* (%) | | A great deal  *n* (%) | |  |
| Lack of time  (*N* = 48) | 22 (45.8) | 14 (29.2) | | 9 (18.8) | | 3 (6.2) | |  |
| Inappropriateness  (*N* = 47) | 7 (14.9) | 23 (48.9) | | 8 (17.0) | | 9 (19.2) | |  |
| Intrusiveness  (*N* = 48) | 7 (14.6) | 18 (37.5) | | 14 (27.1) | | 10 (20.8) | |  |
| Triggering to service users  (*N* = 48) | 11 (29.9) | 12 (25.0) | | 19 (39.6) | | 6 (12.5) | |  |
| Worries about professional boundaries  (*N* = 47) | 14 (29.8) | 10 (21.3) | | 12 (25.5) | | 11 (23.4) | |  |
| Not feeling equipped to help  (*N* = 47) | 9 (19.2) | 8 (17.0) | | 22 (46.8) | | 8 (17.0) | |  |
| Service user vulnerability  (*N* = 47) | 5 (10.6) | 19 (40.4) | | 13 (27.7) | | 10 (21.3) | |  |
| Lack of management support  (*N* = 47) | 22 (46.8) | 8 (17.0) | | 8 (17.0) | | 9 (19.2) | |  |
| Lack of training  (*N* = 47) | 14 (29.8) | 7 (14.9) | | 11 (23.4) | | 15 (31.9) | |  |

**Table 6. Quantitative findings regarding the nature of ‘finding a relationship’ conversations.**

| **Variables** |  | ***n* (%)** |
| --- | --- | --- |
| Proportion of single service users |  |  |
| (Total *N* = 50) | Less than 25% | 6 (12.0) |
|  | 25-50% | 12 (24.0) |
|  | 50-75% | 18 (36.0) |
|  | More than 75% | 14 (28.0) |
| Proportion of single service users wanting to find a relationship |  |  |
| (Total *N =* 50) | Less than 25% | 14 (28.0) |
|  | 25-50% | 16 (32.0) |
|  | 50-75% | 9 (18.0) |
|  | More than 75% | 11 (22.0) |
| Proportion of service users ‘finding a relationship’ conversations are had with |  |  |
| (Total *N* = 50) | None | 5 (10.0) |
|  | Few | 32 (64.0) |
|  | Many | 10 (20.0) |
|  | All, or nearly all | 3 (6.0) |
| Frequency that ‘finding a relationship’ conversations are had  (Total *N* = 50) |  |  |
|  | Never | 5 (10.0) |
|  | Rarely | 26 (52.0) |
|  | Sometimes | 15 (30.0) |
|  | Frequently | 4 (8.0) |
| Who initiates ‘finding a relationship’ conversations  (Total *N =* 50) |  |  |
|  | Always the service user | 16 (32.0) |
|  | Usually the service user | 11 (22.0) |
|  | Sometimes provider, sometimes service user | 16 (32.0) |
|  | Usually provider | 2 (4.0) |
|  | Always provider | 0 (0.0) |
|  | Not applicable | 5 (10.0) |
| Preferred setting for ‘finding a relationship’ conversations  (Total *N* = 57) |  |  |
|  | One to one | 37 (64.9) |
|  | Group | 14 (24.6) |
|  | Unsure | 6 (10.5) |
| Participants who had training in ‘finding a relationship’ support  (Total N = 46) |  |  |
|  | Training | 3 (6.5) |
|  | No training | 43 (93.5) |
